# Modelling the invasion history of *Sinanodonta woodiana* in Europe: Tracking the routes of a sedentary aquatic invader with mobile parasitic larvae

**DOI:** 10.1111/eva.12700

**Published:** 2018-10-20

**Authors:** Adam Konečný, Oana P. Popa, Veronika Bartáková, Karel Douda, Josef Bryja, Carl Smith, Luis O. Popa, Martin Reichard

**Affiliations:** ^1^ The Czech Academy of Sciences Institute of Vertebrate Biology Brno Czech Republic; ^2^ Department of Botany and Zoology Faculty of Science Masaryk University Brno Czech Republic; ^3^ Grigore Antipa National Museum of Natural History Bucharest Romania; ^4^ Department of Zoology and Fisheries Czech University of Life Sciences Prague Prague Czech Republic; ^5^ Department of Ecology & Vertebrate Zoology University of Łódź Łódź Poland; ^6^ School of Biology and Bell‐Pettigrew Museum of Natural History University of St Andrews St Andrews UK

**Keywords:** *Anodonta woodiana*, approximate Bayesian computation, biological invasion, introduction history, invasion genetics, population genetics, unionid mussel

## Abstract

Understanding the invasive potential of species outside their native range is one of the most pressing questions in applied evolutionary and ecological research. Admixture of genotypes of invasive species from multiple sources has been implicated in successful invasions, by generating novel genetic combinations that facilitate rapid adaptation to new environments. Alternatively, adaptive evolution on standing genetic variation, exposed by phenotypic plasticity and selected by genetic accommodation, can facilitate invasion success. We investigated the population genetic structure of an Asian freshwater mussel with a parasitic dispersal stage, *Sinanodonta woodiana*, which has been present in Europe since 1979 but which has expanded rapidly in the last decade. Data from a mitochondrial marker and nuclear microsatellites have suggested that all European populations of *S. woodiana* originate from the River Yangtze basin in China. Only a single haplotype was detected in Europe, in contrast to substantial mitochondrial diversity in native Asian populations. Analysis of microsatellite markers indicated intensive gene flow and confirmed a lower genetic diversity of European populations compared to those from the Yangtze basin, though that difference was not large. Using an Approximate Bayesian Modelling approach, we identified two areas as the probable source of the spread of *S. woodiana* in Europe, which matched historical records for its establishment. Their populations originated from a single colonization event. Our data do not support alternative explanations for the rapid recent spread of *S. woodiana*; recent arrival of a novel (cold‐tolerant) genotype or continuous propagule pressure. Instead, in situ adaptation, facilitated by repeated admixture, appears to drive the ongoing expansion of *S. woodiana*. We discuss management consequences of our results.

## 1 INTRODUCTION

Invasions of non‐native species, defined as a rapid geographical spread and increase in local population size outside the species original range, often threaten native species, communities, and ecosystems (Lockwood, Hoopes, & Marchetti, 2013). Invasive species can cause major ecological and economic problems (Blackburn et al., 2014) and an understanding of what predisposes particular species (and particular populations of those species) to become invasive is one of the central questions in contemporary research in ecology and evolution (Strayer, 2012).

Admixture between individuals from different source populations that meet and interbreed in a non‐native range has been considered as one of the key characteristics driving successful biological invasions (Verhoeven, Macel, Wolfe, & Biere, 2011). Admixture can explain an apparent genetic paradox of invasions when genetically depauperate populations adapt rapidly to a new environment (Roman & Darling, 2007). Admixture ameliorates inbreeding depression (Lallias et al., 2015) and enables mixing of genotypes from a different genetic background and the evolution of novel, transgressive phenotypes (Bock et al., 2015). Admixture also implies a stronger propagule pressure, another outcome that is positively related to invasion success (Lockwood et al., 2013). Adaptive evolution on genetic variation in non‐native populations may also result in strong selection on traits related to invasiveness, perhaps via genetic accommodation of phenotypic plasticity through its exposure in a novel environment (Bock, Kantar, Caseys, Matthey‐Doret, & Rieseberg, 2018).

Freshwater ecosystems are particularly susceptible to biological invasions (Ricciardi & MacIsaac, 2011). Their vulnerability is linked to their high natural and human‐assisted connectivity (Truhlar & Aldridge, 2015), high level of ecological disturbance (Seehausen, van Alphen, & Witte, 1997) generating novel and empty niches (Janáč, Valová, Roche, & Jurajda, 2016), and heavy use for commercial and leisure activities (Anderson, White, Stebbing, Stentiford, & Dunn, 2014). Indeed, some of the most notorious examples of invasive species include examples of freshwater non‐vertebrate (e.g., *Dreissena polymorpha* (Pallas), *Corbicula fluminea* (Muller), and *Procambarus clarki* (Girard)) and vertebrate taxa (*Oreochromis niloticus* (L.), Asian carps) (Nentwig, 2009; Ricciardi & MacIsaac, 2011). Complex life cycles typically decrease the likelihood of a successful invasion, as the invading species needs to find several matching novel environments or biotic partners in the new region (Pichancourt, Chades, Firn, van Klinken, & Martin, 2012). However, an indirect life cycle can also facilitate invasion via a specialized dispersal stage, such as veliger larva in dreissenid mussels, or drifting fish eggs (Chapman et al., 2013; Therriault, Orlova, Docker, MacIsaac, & Heath, 2005).

*Sinanodonta woodiana* (Lea) is a freshwater mussel with a native distribution in East Asia that has invaded several regions outside its native range over the last 50 years. It is a benthic species associated with soft sediment and is known to tolerate low water quality in terms of organic and inorganic pollution (Li et al., 2015). Adult *S. woodiana* combine benthic and filter‐feeding modes (Kim, Lee, & Hwang, 2011) and are highly efficient in depleting sestonic food in the environment (Douda & Čadková, 2017). Female *S. woodiana* brood offspring in their gills and release ripe larvae (glochidia) into the water column where they attach to a fish host to complete development and metamorphose into a free‐living juvenile mussel. Generation time of *S. woodiana* is 2–5 years (Chen, Liu, Su, & Yang, 2015). *S. woodiana* is relatively widespread in its original range, where it inhabits a range of human‐modified habitats such as ponds subject to intensive aquaculture, polluted lakes in urban areas, and irrigation ditches (He & Zhang, 2013). *S. woodiana* has been recorded from Europe (first record in 1979), Indonesia (1969), Dominican Republic (1982), Costa Rica (1994), USA (2010), Myanmar (2016), and Siberia (2016) (Bespalaya et al., 2017; Bogan, Bowers‐Altman, & Raley, 2011; Sárkány‐Kiss, 1986; Vikhrev et al., 2017; Watters, 1997).

While detected in the wild in Europe in 1979 in western Romania (Sárkány‐Kiss, 1986) and in southern France in 1982 (Adam, 2010), the species may have been present in aquaculture facilities in Eastern Europe since 1959 (Watters, 1997). Two potential sources of *S. woodiana* are recognized. First, juvenile Asian carp were introduced to Romania from the River Yangtze basin in China in 1959 and 1962. Second, Asian carps were imported to Hungary from the River Amur basin in 1963–1965. In both cases, imported fish were putatively infected with *S. woodiana* glochidia that established adult mussel populations in hatcheries (reviewed in Watters, 1997). After an isolated record from France in 1982 with a trace to the Hungarian source (Adam, 2010), local establishment in southern Hungary (1988) and in a system of artificially heated lakes in central Poland (1993), the species appeared to show no further signs of dispersal and it was long considered a thermophilic species (Kraszewski & Zdanowski, 2001) with low invasive potential. However, *S. woodiana* started to spread in the first decade of the 21st Century (Lajtner & Crnčan, 2011) and its populations are now recorded from much colder habitats, including subalpine lakes in northern Italy (2010) (Kamburska, Lauceri, & Riccardi, 2013) and regions that are subject to relatively long winters (southern Sweden in 2005) (Svensson & Ekström, 2005). At least two European populations of *S. woodiana* possess separate morphotypes on the basis of their shell shape, with a distinct coevolutionarily driven response to a parasitic fish in Europe (Reichard et al., 2015). A recent study of two Italian *S. woodiana* populations demonstrated that the two morphotypes are not genetically distinct at a mitochondrial marker (Guarneri et al., 2014), suggesting that morphological variability of *S. woodiana* populations in Europe represents a plastic response to environmental conditions (a common feature of unionid mussels) rather than genetically determined variability (Soroka & Zdanowski, 2001). Yet, the genetic variability of European *S. woodiana* populations beyond mitochondrial markers is unknown, despite a potential for population‐specific impacts of invasive *S. woodiana* populations on native communities and ecosystems.

In the present study, a set of 16 populations collected from the European non‐native range of *S. woodiana* was used to investigate the origin and dispersal of *S. woodiana* in Europe, using a mitochondrial marker (fragment of the *COI* gene) and a set of specifically designed nuclear microsatellite markers (Popa, Bartáková, Bryja, Reichard, & Popa, 2015; Popa et al., 2011). European *S. woodiana* populations were compared with genetic variability in the native range of the species in East Asia. The main aims of the study were as follows: (a) to describe the genetic diversity and structure of invasive *S. woodiana* populations in Europe; (b) to identify the putative original European populations that served as sources for the subsequent invasion across the continent; (c) to model complex associations across the non‐native European range, including the level of admixture and its spatial structure, using Approximate Bayesian Computation; (d) to locate the potential sources of *S. woodiana* from its native range that were introduced to Europe, and (e) to consider the management implications of the results.

## 2 MATERIALS AND METHODS

### 2.1 Sampling

**Figure 1:**
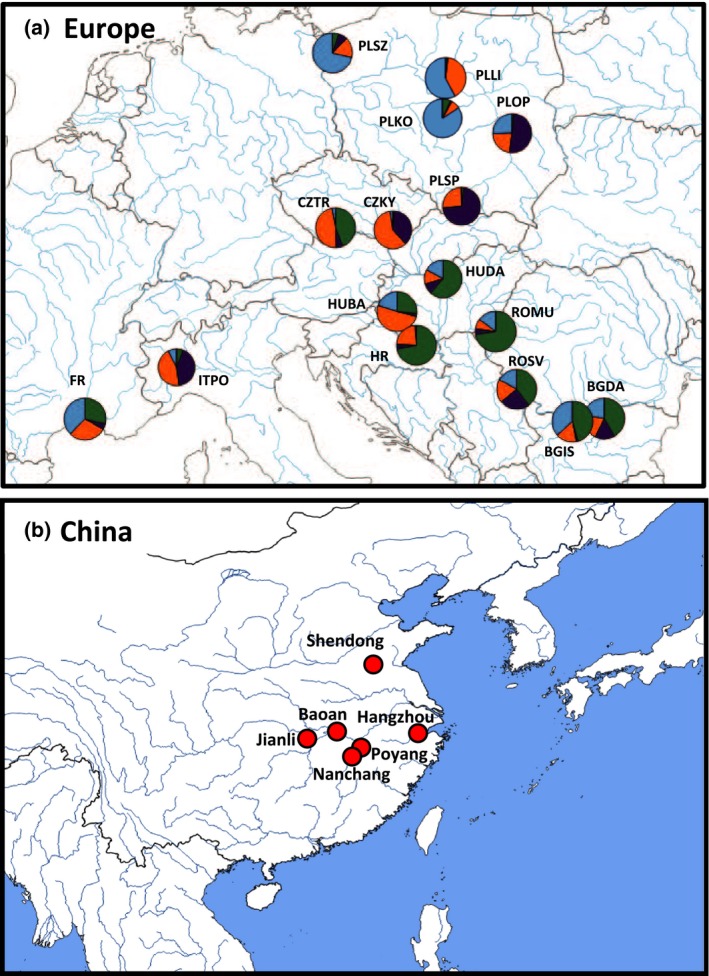
Geographic distribution of all European (a) and Chinese (b) populations of *S. woodiana* analyzed at microsatellite markers. (a) Pie chart colors illustrate microsatellite‐marker genetic variation represented by proportional membership of individuals to microsatellite‐based clusters for *K* = 4. Sample codes are summarized in Table 1. (b) Red circles illustrate geographic position of analyzed populations in their native range

Samples of *S. woodiana* were collected in Europe (non‐native range) and Asia (native range) between 2011 and 2014 by the authors and collaborators (Table 1). We sequenced the *COI* region of the mitochondrial genome in 22 individuals from two Asian populations and 40 individuals from nine European populations. To analyze the detailed invasion history of the species in Europe, we genotyped 369 *S. woodiana* individuals from 16 populations in Europe and 137 individuals from six populations in China (Table 1; Figure 1). Our sampling included the regions where *S. woodiana* was first recorded in Europe; the population ROMU was located 85 km from the first record of *S. woodiana* outside an aquaculture facility in Europe in 1979 (Cefa, Bihor County, Romania) (Sárkány‐Kiss, 1986), the population FR was collected close to Arles (France), where *S. woodiana* shells were reported in 1982 as the second oldest record in Europe (Adam, 2010), and a group of populations from Poland (PLSZ, PLLI and PLKO) was from a region where *S. woodiana* was known since 1993 (Soroka, 2000). Mussels were sampled by visual and tactile searching of stream and lake substrates. A piece of mantle tissue was excised from each mussel (Berg, Haag, Guttman, & Sickel, 1995), and the tissue samples (or entire specimens) were preserved in 95% ethanol in the field and subsequently stored at −80°C prior to DNA extraction.

**Table 1:** Sampling site information and genetic diversity of *S. woodiana* in its non‐native (Europe) and native (China) range

Site	Code	Country	Lat	Long	N ind	N allele	AR_6_	AR_17_	*H*_O_	*H*_E_	*F*_IS_
Europe											
Danube	BGDA	Bulgaria	43.672	25.637	30	8.2	4.92	7.46	0.733 ± 0.165	0.778 ± 0.103	0.059
Iskar	**BGIS**	Bulgaria	43.585	24.359	18	6.9	4.80	6.85	0.797 ± 0.129	0.781 ± 0.105	−0.022
Svinita	**ROSV**	Romania	44.496	22.099	22	7.9	4.69	7.38	0.786 ± 0.128	0.775 ± 0.093	−0.014
Mures	**ROMU**	Romania	46.149	21.248	9	6.4	4.82	–	0.799 ± 0.179	0.761 ± 0.113	−0.054
Drava	HR	Croatia	45.764	18.049	29	8	4.60	7.05	0.729 ± 0.113	0.773 ± 0.069	0.058
Danube	HUDA	Hungary	47.621	19.106	8	4.7	4.22	–	0.798 ± 0.131	0.746 ± 0.133	−0.076
Balaton	HUBA	Hungary	46.711	17.254	21	7.5	4.61	7.07	0.776 ± 0.136	0.764 ± 0.072	−0.016
Kyjovka	**CZKY**	Czech R.	48.940	17.072	63	8.1	4.22	6.35	0.715 ± 0.184	0.706 ± 0.156	−0.013
Trebonsko	**CZTR**	Czech R.	48.990	14.748	10	5.8	4.70	–	0.788 ± 0.207	0.787 ± 0.053	−0.002
Szczecin	**PLSZ**	Poland	53.442	14.604	20	7.6	4.54	7.28	0.750 ± 0.181	0.727 ± 0.140	−0.033
Konin	**PLKO**	Poland	52.289	18.240	25	7.6	4.39	6.86	0.781 ± 0.135	0.732 ± 0.090	−0.068
Lichen	**PLLI**	Poland	52.301	18.319	36	8.1	4.78	7.05	0.758 ± 0.166	0.774 ± 0.092	0.021
Opatkowice	**PLOP**	Poland	51.454	21.882	34	7	4.27	6.10	0.682 ± 0.127	0.733 ± 0.097	0.070
Spitkowice	**PLSP**	Poland	49.576	19.843	28	6.3	4.06	5.81	0.650 ± 0.265	0.677 ± 0.184	0.040
Po	ITPO	Italy	45.149	8.331	10	4.9	3.97	–	0.650 ± 0.280	0.676 ± 0.200	0.040
Arles Rhône	**FR**	France	43.656	4.600	6	5.5	5.11	–	0.713 ± 0.221	0.795 ± 0.128	0.111
China											
Shendong	CNSH	China	34.744	117.146	11	6.7	4.90	–	0.746 ± 0.178	0.603 ± 0.216	0.200
Nanchang	CNNA	China	28.665	115.815	27	13.7	6.37	11.73	0.868 ± 0.084	0.812 ± 0.114	0.063
Lake Bao'an	CNBA	China	30.239	114.727	27	13.8	6.25	11.86	0.842 ± 0.124	0.792 ± 0.102	0.061
Hangzhou	CNHA	China	30.251	120.145	24	11.7	5.59	10.27	0.764 ± 0.228	0.644 ± 0.260	0.165
Jiali	CNJI	China	29.792	112.877	24	8.2	4.59	7.45	0.710 ± 0.175	0.708 ± 0.208	0.002
Lake Poyang	CNPO	China	29.165	116.225	24	13	6.29	11.87	0.853 ± 0.112	0.700 ± 0.180	0.183

*Notes*

Sample sites with codes in bold were used in the ABC analyses.

*AR*_6_: mean allelic richness calculated for 6 individuals; *AR*_17_: mean allelic richness calculated for 17 individuals; *F*_IS_: inbreeding coefficient; *H*_E_: expected heterozygosity (with their standard deviations); *H*_O_: observed heterozygosity (with their standard deviations); Lat: Latitude, Long: Longitude; N allele: the mean number of alleles; N ind: the number of sampled individuals.

### 2.2 DNA extraction and genotyping

Genomic DNA was extracted using the NucleoSpin^®^ Tissue kit. A 370 bp fragment of the mitochondrial gene for cytochrome oxidase c subunit I (*COI*) was amplified using the primers LCO1490 and HCO2198 (Vrijenhoek, 1994). A set of 17 microsatellite loci developed specifically for *S. woodiana* (Popa et al., 2015) was genotyped using the Qiagen Multiplex PCR kit (QiagenTM). Alleles were scored in GENEMAPPER v. 5.0 (Applied Biosystems, Foster City, USA) and double‐checked manually. The presence of null alleles for each locus and population was assessed with FreeNA (Chapuis & Estoup, 2007). Detailed protocols are available in Supporting Information (Appendix S1).

### 2.3 Mitochondrial data analysis

A total of 127 *COI* sequences were analyzed (62 produced in this study and 65 retrieved from GenBank; 78 from Europe and 49 from Asia) (Supporting Information Table S1). Genetic diversity within populations and within continents (i.e., native and non‐native ranges) was estimated as haplotype diversity (*Hd*; a probability that two randomly sampled alleles are different) and nucleotide diversity (*Pi*; average number of nucleotide differences per site in pairwise comparisons among sequences) (Nei, 1987), calculated in DnaSP 5.10.1 (Librado & Rozas, 2009). Sequence variation was visualized as a haplotype network using the median‐joining algorithm in Network 5.0.1 (Bandelt, Forster, & Röhl, 1999). The number of base differences per site between groups of sequences was computed in MEGA6 (Tamura, Stecher, Peterson, Filipski, & Kumar, 2013).

### 2.4 Genetic diversity and structure

Of the 17 originally developed loci, a high frequency of null alleles (mean >0.07 per population) was detected at seven loci and they were excluded from all subsequent analyses to avoid the biases caused by null alleles such as an increase in interpopulation divergence (Chapuis & Estoup, 2007). Genetic diversity was estimated over the remaining 10 loci by calculating observed heterozygosity (*H*_O_) and unbiased expected heterozygosity according to Nei (1978) (*H*_E_) in GENETIX 4.05.2 (Belkhir, Borsa, Chikhi, Raufaste, & Bonhomme, 1996‐2004). Mean allelic richness (*A*_R_) over loci was determined with the rarefaction procedure in FSTAT 2.9.3 (Goudet, 2001) to estimate the expected number of alleles in subsamples of six individuals (corresponding to the smallest population sample). Additionally, the expected number of alleles was estimated for a subsample of 17 individuals (populations with less individuals were excluded from this analysis). Pairwise *F*_ST_ values for 16 European and six Chinese populations were calculated with FSTAT. The concordance between genetic (linearized *F*_ST_ according to Slatkin, 1995) and geographic distances (Euclidian distances in km) among European populations (isolation by distance) was analyzed using a Mantel test (999 permutations) in GenAlEx 6.501 (Peakall & Smouse, 2012). Linear geographic distances were used because of an a priori expectation that long‐distance dispersal (between river basins) arose from human‐mediated transport rather than natural dispersal on fish hosts. Differences in genetic diversity (allelic richness, the unbiased estimate of the gene diversity (*GD*: Nei, 1978) and *F*_ST_ between the introduced European and native Chinese populations were tested using a two‐sided permutation test (1,000 permutations) implemented in FSTAT.

The spatial genetic structure of European populations was investigated with the Bayesian approach implemented in STRUCTURE version 2.3.4 (Pritchard, Stephens, & Donnelly, 2000). First, we analyzed native and non‐native populations in a single analysis. This analysis provided a clear distinction between native and non‐native populations at *K *=* *2, with no mixing between Chinese and European populations at higher *K* values (Supporting Information Figure S1). We, therefore, ran a second analysis, in which, only samples from non‐native European range were included, because we were primarily interested in the relationship between populations in the non‐native range. In that analysis, we assumed an admixture model, in which the algorithm assigns proportions of individual genotypes to each of the clusters. We performed 20 independent runs for each *K* value (from 1 to 11), with different values of the Dirichlet *α* parameter for each assumed cluster. We used a model with correlated allele frequencies (Falush, Stephens, & Pritchard, 2003) and with sampling locations as prior information to assist clustering (i.e., LOCPRIOR model sensu Hubisz, Falush, Stephens, & Pritchard, 2009). Each run included 300,000 burn‐in iterations followed by 600,000 iterations.

Post‐processing of the STRUCTURE output was performed with the CLUMPAK software (Kopelman, Mayzel, Jakobsson, Rosenberg, & Mayrose, 2015) to infer pairwise similarity between Q‐matrices for each *K*. We used the LargeKGreedy algorithm, random input order, and 2,000 repeats. We identified different modes from the results of the 20 runs for each *K* value at a threshold of 0.9 for similarity scores. We averaged individual membership proportions for all runs in the same mode and produced summary bar plots for a given *K* value. We displayed the probability of the data (ln *p*(*K*|*D*)) for each *K* value.

### 2.5 Inference of invasion pathways in Europe by approximate Bayesian computation

For the analysis of invasion pathways in an Approximate Bayesian Computation (ABC) framework (Beaumont, Zhang, & Balding, 2002), we selected samples representing each group of genetically coherent samples, which were defined as samples that hold coherent STRUCTURE assignments across several *K* values. When two (three cases: BGIS and BGDA, CZTR and HUBA, PLOP and ITPO) or three (one case: ROMU, HR, and HUDA) populations formed a single group, only one population was chosen as the representative sample. This resulted in the selection of 11 populations (sampling sites) for ABC analysis (Table 1).

The relationship among the 11 populations was modelled by comparing posterior probabilities for various models (i.e., evolutionary scenarios), using DIYABC 2.0.4 (Cornuet et al., 2014). This coalescent‐based method enables the definition and comparison of a set of demographic and evolutionary scenarios with the aim of explaining the evolutionary history and relationships among contemporary populations. DIYABC generates simulated datasets for each scenario and compares selected summary statistics of the real dataset to those of simulated datasets to calculate the posterior probabilities for each tested scenario.

In the absence of a robust a priori hypothesis for the invasion history of *S. woodiana* in Europe, and given high admixture, we used an exploratory ABC approach. We modelled relationships (origin) within each pair of populations by building 55 independent “pairwise ABC comparisons,” an approach similar to Miller et al. (2005) on population triplets. Each comparison was based on the same set of competing models (evolutionary scenarios), the same set of microsatellite markers, and the same prior distribution of parameters. The most likely scenario was always inferred from a set of 12 models (i.e., possible scenarios) for each pair of populations, differing in the identity of the source populations and the presence of admixture (Supporting Information Figure S2). The population pair (populations labelled as F = “first” and S = “second” in Supporting Information Figure S2 and Table S2) may have originated from the following sources: (a) both F and S from the same ancestral (A) population (scenario 1); (b) F from S and vice versa (scenarios 2 and 3); (c) F from A, S from an unsampled invasive population (U) derived from A, and vice versa (scenarios 4 and 5); (d) both F and S from U derived from A (scenario 6); (e) F derived through an admixture event between S and U, also originating from S, and vice versa (scenarios 7 and 8); (f) F derived through an admixture event between S and U originating from A and vice versa (scenarios 9 and 10); and (g) F derived through an admixture event between U derived from A and the second population in a pair (S) originating from the same source (U) and vice versa (scenarios 11 and 12). The prior probability of data simulation under each of these 12 scenarios was equal (i.e., 0.0833).

Each scenario was defined by a combination of three demographic events (change in effective population size, population split, and merging of two populations). Resulting simulated genotypes (represented by their summary statistics) were compared to the sampled genotypes. Each event was linked to particular parameters: timing of the event (generations to the past), effective population size, or admixture rate (i.e., the genotypic proportion of ancestral populations when merging them into a single derived population). In addition, because we modelled invasion events, we always considered a potential bottleneck effect in each newly founded population (defined by the duration of bottleneck and limited effective population size during the time of the bottleneck). The definition of the 12 scenarios in the DIYABC is reported in Supporting Information Figure S2. The prior distributions of the parameters were uniform, and their ranges were as follows: effective population sizes 10–20,000 individuals, timing of events 2–100 generations ago, admixture rate 0–1, bottleneck duration 0–20 generations, and effective population size during bottleneck (i.e., the number of founders) 2–500. Both ancestral and unsampled populations were “ghost” populations, that is, neither of them was sampled. Given that the ABC analysis does not take geographic information into account, we were able to distinguish between ancestral and unsampled populations using different temporal conditions, with any split from A being considered, a priori, older than all other subsequent events (for the list of conditions between parameters see Supporting Information Figure S2).

The DIYABC analyses were conducted with two groups of loci: The first group included five loci with a dinucleotide microsatellite motif (SW_13, SW_14, SW_15, AW_28, and AW_570) and the second group was composed of five loci with a trinucleotide motif (AW_521, AW_238, AW_292, AW_324, and AW_514). Generalized Stepwise Mutation models (Estoup, Jarne, & Cornuet, 2002) were run for each group (dinucleotide and trinucleotide motifs) separately, using the same priors. We set default values for microsatellites in DIYABC, except the mean mutation rate, where we used a larger uniform prior interval from 10^−5^ to 10^−3^. To estimate sensitivity to priors, we reran the same 55 pairwise comparisons with log‐uniform prior distributions (over the same range as for uniform priors) for effective population sizes and timing of events. For model choice inference, we generated a table of 10^6^ simulated datasets for each of the 12 scenarios for each of the ABC pairwise comparisons.

The ABC method is based on summary statistics calculated from the data to represent the maximum amount of information in the most parsimonious form. The within‐ and between‐population genetic variation of the populations (independently for both groups of microsatellites) was summarized with the statistics equivalent to those traditionally used in population genetics); NAL: mean number of alleles, HET: mean genic diversity, N2P: mean number of alleles in two samples, FST, LIK: classification index, and DM2: (*dμ*)^2^ distance (Cornuet et al., 2014). We compared the different scenarios by calculating their relative posterior probabilities by polychotomous logistic regression (Cornuet et al., 2008) from the 1% of simulated data sets most closely resembling the observed data set (in a multidimensional space of the summary statistics) in terms of the calculated discriminant scores within the option “Linear discriminant analysis on summary statistics” included in DIYABC by Estoup et al. (2012).

Selection of the most likely evolutionary scenarios for each “ABC pairwise comparison” was based on the highest relative posterior probability with 95% credible intervals (CI) not overlapping with those for the other scenarios. Accordingly, a single or several scenarios could be selected for each “pairwise comparison.” We compared the outcome of the analyses with both prior settings and selected scenarios that were supported in both analyses. We assessed the goodness‐of‐fit of the best model by evaluating consistency of the observed data with the posterior predictive distribution of the model for the best scenarios. We conducted model checking for all 55 pairwise comparisons using all summary statistics, including those that had not been used in the initial ABC analyses for model selection (i.e., VAR: mean allele size variance, MGW: mean M index, H2P: mean gene diversity in two samples, V2P: mean allele size variance in two samples, and DAS: shared allele distance) (Cornuet, Ravigne, & Estoup, 2010; Cornuet et al., 2014). Finally, using a new analytical tool in the latest version of DIYABC (v 2.1.0), we calculated a “posterior” error rate to estimate accuracy in model choice conditional on the observed dataset (i.e., focusing around the observed data set by using the posterior distributions of the scenario ID and parameters). In this analysis, we simulated 100 pseudo‐observed data sets drawn randomly from the 500 simulated data sets closest to the observed dataset (in the multidimensional space of the summary statistics).

## 3 RESULTS

### 3.1 Mitochondrial diversity

**Figure 2:**
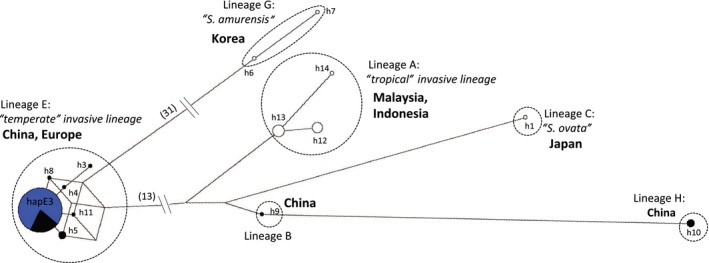
Haplotype network of all 127 *S. woodiana* COI sequences retrieved from GenBank and analyzed for the current study. Five major mitochondrial haplogroups were named in accordance with Bolotov et al. (2016). Blue represents European samples, and black indicates samples from China (Yangtze basin)

An alignment of partial *COI* sequences (370 bp) obtained from 78 individuals from Europe and 49 individuals from Asia (Supporting Information Table S1) contained 54 polymorphic sites and produced a total of 14 haplotypes. Haplotypes grouped into five divergent haplogroups (Figure 2). All European samples (including two individuals from Arles, France) contained a single haplotype (E3 sensu Vikhrev et al., 2017) that clustered with samples from China (middle Yangtze basin, including Lake Poyang). Other haplogroups contained samples from different Asian regions, including China (Lake Poyang). Samples from the River Amur basin clustered to a different haplogroup (Figure 2). Given the unresolved taxonomy of *S. woodiana* (e.g., Vikhrev et al., 2017; see Discussion) and our focus on invasive populations in Europe, we only analyzed the mtDNA diversity of the haplogroup present in Europe (i.e., Lineage E in Figure 2) in detail (106 sequences from 78 European and 28 Chinese samples). Within this haplogroup, we identified only five polymorphic sites, defining six closely related haplotypes (Figure 2). Five haplotypes were endemic to China (the Yangtze basin), while one haplotype was co‐distributed in Europe and the River Yangtze basin. The single haplotype of European *S. woodiana* (E3) was also the most common Asian haplotype (Figure 2).

### 3.2 Microsatellite genetic diversity

Genetic variability over 10 microsatellite loci in 369 individuals from 16 European populations was two to 16 alleles per locus and population (mean 6.9), lower than in Chinese populations (three to 21 alleles per locus and population, mean: 11.1). Standardized mean allelic richness (AR_6_) varied from 3.97 in Italian population (ITPO) to 5.11 in a population from southern France (FR) and the pattern of allelic richness for AR_17_ was concordant. The expected unbiased heterozygosity over all loci (*H*_E_) ranged from 0.676 ± 0.200 (*SD*) to 0.795 ± 0.128 in ITPO and FR, respectively (Table 1).

Genetic diversity in six Yangtze basin populations was larger than in the introduced European populations (allelic richness AR_6_: 5.663 vs. 4.543, two‐sided permutation test in FSTAT, *p *<* *0.001; gene diversity *GD*: 0.806 vs. 0.743, *p *=* *0.022). However, there was no significant difference between *F*_ST_ within European and Yangtze populations (two‐sided permutation test, *p *=* *0.654), with mean *F*_ST_ of 0.054 in Europe and 0.068 in the Yangtze basin. In Europe, mean pairwise genetic differentiation between populations ranged from 0.005 (FR vs. BGDA) to 0.144 (PLSP vs. ROMU; Supporting Information Table S3). Likewise, observed heterozygosity (*H*o) did not differ between populations from Europe (0.736) and the Yangtze basin (0.726) (*p *=* *0.677).

### 3.3 Microsatellite genetic structure in Europe

**Figure 3:**
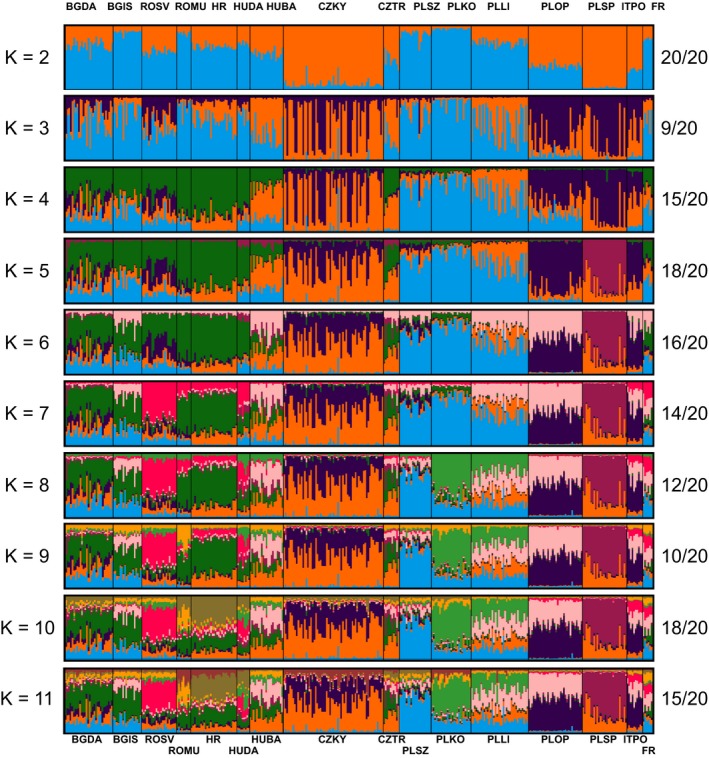
STRUCTURE output for *K* of 2–11 for 20 replicate runs based on 10 microsatellite markers and 369 individuals from 16 European populations of *S. woodiana* summarized with CLUMPAK. For *K* = 3, only mode A is presented. Proportion of independent STRUCTURE runs generating the results presented are provided on the right. For sample codes, see Table 1

A Mantel test indicated a lack of isolation by distance structure among European populations (Mantel's *r *=* *−0.137, *p *=* *0.231). Bayesian clustering analysis revealed weak population genetic structure in our dataset but with populations of relatively consistent genetic background across an increasing number of putative clusters (Figure 3). Separation into four clusters (*K *=* *4; the highest *K* whose runs had relatively similar probability, Supporting Information Figure S3) demonstrated a coherent group of populations from south‐eastern Europe (green) and a group of populations from central and northern Poland (light blue) (Figure 1a). Importantly, all populations remained largely admixed regardless of the number of clusters (Figure 3), although some populations formed consistently coherent groups, irrespective of the number of clusters (PLOP and ITPO; CZTR and HUBA; ROMU, HR and HUDA; BGIS and BGDA; Figure 3, Supporting Information Figure S4).

### 3.4 Inference on invasion pathways in Europe

**Figure 4:**
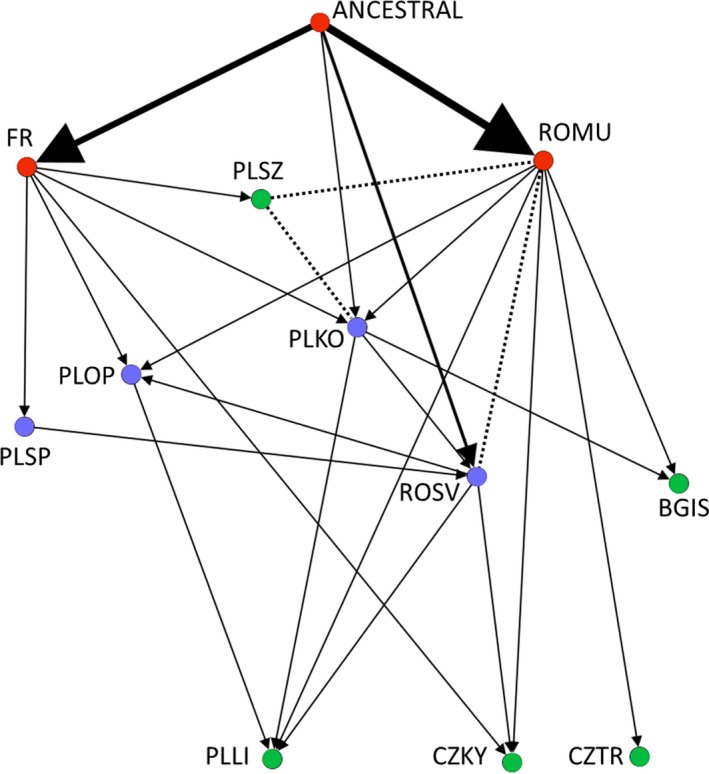
The most likely colonization pathways (indicated by arrows) of *S. woodiana* in Europe inferred by ABC pairwise comparisons between 11 populations and a putative ancestral source. The relationships are derived from the single‐winner scenarios and two cases of the double‐winner scenarios. The relationships are supported by admixture scenarios, demonstrating that a population was cofounded from the population indicated by arrows and another (non‐identified) population (summarized in Table 2). Red nodes refer to the source populations, blue nodes represent intermediate populations, and green nodes indicate derived populations. The thickness of lines leading from ANCESTRAL is weighted to indicate the strength of support. Dotted lines connect population samples whose pairwise ABC comparison resulted in multiple scenarios being equally supported. Sample codes are given in Table 1

Eleven populations selected for ABC analysis were chosen to represent the genetic and geographic variability of *S. woodiana* in Europe (Table 1, Figure 3). From the total of 55 ABC analyses, 31 (56%) analyses identified a single best model (i.e., the model with the highest relative posterior probability and 95% CI not overlapping with the 95% CI of the other models in the analysis; Supporting Information Table S4). They are referred to as “single‐winner” pairwise comparisons. The two best models had overlapping CIs in their posterior probabilities in 14 pairwise analyses (26%; “double‐winner” comparisons) and 10 pairwise comparisons had three or more best models with overlapping CIs. Three pairwise comparisons (ROMU vs. ROSV, PLSZ vs. ROMU, and PLKO vs. PLSZ; dotted edges in Figure 4) failed to identify any well‐supported model, with most scenarios possessing similar relative posterior probabilities (Supporting Information Tables S2 and S4). This outcome indicates that there was not sufficient signal for a clear genetic distinction to be drawn between members of these three pairs and those populations were likely closely related. Confidence in scenario choice and robustness to changes in prior distribution of parameters was largely confirmed by an analysis with log‐uniform priors, with 38% (17 of 45) of pairwise contrasts with a “single‐winner” and “double‐winner” scenarios entirely concordant between the analyses with different priors, and 89% (40 of 45) pairwise comparisons sharing at least one of the two supported scenarios (Supporting Information Table S5).

The following results (summarized in Figure 4 and Supporting Information Figure S5) are based on unambiguously supported and informative (i.e., demonstrating a direct genetic contribution within a population pair or from an ancestral population) scenarios in the 20 analyses; 18 single‐winner comparisons and two double‐winner comparisons that provided concordant results (bold in Supporting Information Table S2) and corresponded between the two prior sets. The graphical representation of the assessment of the goodness of fit of the best model (model checking) for each of the single‐winner comparisons demonstrated that the observed dataset had fallen well within the cloud of the simulated parameter estimates for the first four principal components (PCA plots in Supporting Information Figure S6). Posterior error rates for each pairwise comparison are provided in Supporting Information Table S6.

The relationships among 11 sampled European *S. woodiana* populations separated them into three categories. (a) Sampled “source” populations in Europe (ROMU and FR; red in Figure 4) were always derived from an ancestral population (A) and cofounded numerous other populations. A direct origin from A was also suggested in two (of six) analyses of ROSV (located close to ROMU) and one (of nine) single‐winner comparisons of PLKO (Table 2, Supporting Information Table S2). (b) “Derived” populations (BGIS, CZKY, CZTR, PLSZ, and PLLI; green in Figure 4) had no single‐winner comparison that identified them as the source of any other sampled population. (c) “Intermediate” populations (ROSV, PLKO, PLOP, and PLSP; blue in Figure 4) served both as descendants and as sources of other populations. Notably, the scenario where one of the two compared populations was unique (i.e., without admixture), the direct source of the second population (scenarios 2 or 3; Table 2 and Supporting Information Table S2) had never been selected as a single‐winner comparison (Figure 4). This finding implies frequent gene flow among European populations of *S. woodiana* and is further supported by the fact that 17 (55%) of the best supported scenarios in the single‐winner pairwise comparisons involved an admixture event between another sampled population and an unsampled population (scenarios 7–12; Supporting Information Table S2). In two pairs, both top scenarios in the double‐winner comparisons indicated a concordant relationship; that is, PLSZ was cofounded from FR and PLLI was cofounded from ROMU (Tables 2 and Supporting Information Table S2). The ABC analysis demonstrates that FR and ROMU, two “source” populations for further colonization of Europe, were not derived from two independent introductions from the native range but rather represent a single colonization event (Supporting Information Tables S2 and S4: strongly supported scenarios 2, 7, 11 and 12 and no support for scenarios 1, 4, 5, 9 and 10).

**Table 2:** Number of single‐winner pairwise comparisons for each of 11 tested populations of *S. woodiana*

Population	Source: ancestral	Source: admixture^a^	Source: unsampled	N_SingleWin_
BGIS	–	2: ROMU, PLKO	4	6
ROSV	2	2: PLKO, PLSP	2	6
ROMU	5	–	–	5
CZKY	–	3: ROSV, ROMU, FR	3	6
CZTR	–	1: ROMU	4	5
PLSZ	–	(+1: FR)	2	2
PLKO	1	2: ROMU, FR	6	9
PLLI	–	3: ROSV, PLKO, PLOP (+ ROMU)	3	6
PLOP	–	3: ROSV, ROMU, FR	5	8
PLSP	–	1: FR	4	5
FR	4	–	–	4

*Notes*

The source of the *S. woodiana* population sample is indicated. When admixture scenario was supported, the co‐source populations are listed. Results in parentheses are based on concordant relationships from two supported scenarios in double‐winner comparisons.

N_SingleWin_: number of single‐winner comparisons.

a Admixture from sampled + unsampled population.

## 4 DISCUSSION

We have documented largely depauperate mitochondrial diversity in *S. woodiana* in non‐native European populations, in contrast to large mitochondrial genetic variability of *S. woodiana* in its native range in Asia. A single haplotype detected in all European samples matched the most common haplotype present in the River Yangtze basin in China. Nuclear microsatellite markers indicated a high level of admixture among European populations, consistent with intensive gene flow and no pattern of isolation by distance. Genetic diversity of microsatellite markers was lower in Europe than in populations sampled from the Yangtze basin, though the decrease was not dramatic and genetic differentiation and observed heterozygosity did not differ between Europe and the Yangtze basin. ABC modelling recovered two areas (southern France and the region of western Romania, southern Hungary and northern Croatia), geographically concordant with the oldest European records, as the source of the other *S. woodiana* populations in Europe, a sets of derived populations (current invasion endpoints) and populations that were historically derived from other non‐native populations and subsequently served as a source of further invasion.

In the wild, *Sinanodonta woodiana* was first recorded in Europe in 1979 in western Romania (middle Danube basin) (Sárkány‐Kiss, 1986), followed by a record of a shell in southern France near Arles (the lower river Rhone basin) in 1982 (Adam, 2010). Its initial spread was slow and restricted to artificially heated waters (Urbanska, Lakomy, Andrzejewski, & Mazurkiewicz, 2012). However, the species became widespread in Europe during the first two decades of the 21st Century (Lopes‐Lima et al., 2017), colonizing much of continental Europe, invading as far north as Sweden (Svensson & Ekström, 2005) and including all three southern peninsulas (Lajtner & Crnčan, 2011; Pou‐Rovira et al., 2009; Solustri & Nardi, 2006). This rapid expansion by *S. woodiana* is well supported by our population genetic data. The presence of an identical *COI* haplotype indicates an origin from a single source region. The possibility of colonization from two different source regions (the Amur and Yangtze basins which possess largely divergent mitochondrial lineages (Sayenko, Soroka, & Kholin, 2017)), as suggested by historical records of fish vector imports (Adam, 2010; Watters, 1997), was not supported (Figure 2; Supporting Information Figure S5). Microsatellite data indicated a single colonization event and an early establishment of two invasive centers serving as sources for further expansion across Europe. This information suggests that a commercial import of Asian carps from the River Yangtze basin to hatcheries in Romania in the early 1960s was the most likely source of further *S. woodiana* expansion in Europe. Imports of *S. woodiana* to Hungarian hatcheries most likely involved *S. woodiana* populations from the River Amur basin (reviewed in Watters, 1997), possessing *COI* haplotypes that are not present in Europe.

*Sinanodonta woodiana* was localized in largely discrete regions of Europe until the early 21st Century, when its rapid expansion through the continent became manifest (Lajtner & Crnčan, 2011), following a typical demographic and temporal pattern seen in many invasive species (Sakai et al., 2001). Originally considered as a thermophilic species (Kraszewski & Zdanowski, 2007; Spyra, Jedraszewska, Strzelec, & Krodkiewska, 2016) with limited invasion potential, *S. woodiana* has now colonized habitats with low water temperatures (Kamburska et al., 2013). It is possible that overcoming a thermal limitation in its reproduction was the evolutionary innovation that triggered the invasion of *S. woodiana* across Europe (Douda, Vrtílek, Slavík, & Reichard, 2012; Galbraith & Vaughn, 2009; Kraszewski, 2007). It appears that novel (cold‐tolerant) phenotypes arose through in situ adaptation and were perhaps facilitated by repeated admixture. Genetic accommodation of existing phenotypic plasticity driven by adaptive evolution on reaction norms has recently been implicated in the evolution of invasiveness (Bock et al., 2018) and this scenario fits the patterns observed in our analyses. There was no indication of recent or repeated introduction of new genotype(s) to support the hypothesis of recent arrival of a novel (cold‐tolerant) genotype or intensive propagule pressure from native populations driving admixture and subsequent expansion of *S. woodiana*.

The rapid spread of *S. woodiana* was likely facilitated by a life cycle that involves a parasitic stage (termed a glochidium). Glochidia are released into the water column and attach to a fish host to complete development. Glochidia of *S. woodiana* remain attached to the gills or fins of the host fish for 5–20 days (Donrovich et al., 2017) before metamorphosing into a free‐living juvenile mussel. That period is sufficient for successful long‐distance dispersal associated with the trade in freshwater fishes for aquaculture and angling purposes across Europe (Litvak & Mandrak, 1999). While many unionid mussel species are host specialists (Modesto et al., 2017), *S. woodiana* has an extremely extensive host range and can utilize all European freshwater fish species hitherto tested (Douda et al., 2012, 2017). This feature is not unique to invasive *S. woodiana* populations, because *S. woodiana* also utilizes an exceptionally broad range of host species in its native range (Douda et al., 2017; Dudgeon & Morton, 1984). This trait is a highly effective preadaptation for rapid and successful invasion (Torchin & Mitchell, 2004). It appears that the combination of a generalist host utilization and the widespread commercial trade in freshwater fishes has contributed to the rapid invasion of *S. woodiana* across Europe, as well as in other regions of the world (Lajtner & Crnčan, 2011; Vikhrev et al., 2017; Watters, 1997).

All sampled *S. woodiana* populations in Europe possessed a single mitochondrial haplotype. This haplotype was common in the River Yangtze basin in China, at the center of the natural distribution of *S. woodiana*. This finding points toward the potential source region of *S. woodiana* in Europe being in Central China, a region that is a frequent source of invasions to the western Palearctic (Nentwig, 2009). *S. woodiana* has a relatively wide natural distribution, from temperate southeast Russia (River Amur basin) to tropical southern China, with large genetic divergence among lineages (Vikhrev et al., 2017). Given its more compatible climate, the River Amur basin is another potential source of *S. woodiana* in Europe (Watters, 1997), particularly given the well‐documented historical commercial links between that region and Eastern Europe (Watters, 1997). However, the region is home to populations of *S. woodiana* with distinctly different mitochondrial lineages to those in Europe (Bespalaya et al., 2017; Sayenko et al., 2017). Similarly, *S. woodiana* populations on the Korean Peninsula and from Japan are genetically distant from European populations (Vikhrev et al., 2017) and invasive populations of *S. woodiana* in south‐eastern Asia belong to a different mitochondrial haplogroup (“tropical invasive lineage A” sensu Vikhrev et al., 2017). Recently (in 2016), a population possessing the identical haplotype to all European *S. woodiana* populations has been discovered in Myanmar, though it is not yet clear whether this population has invasive potential in the region (Vikhrev et al., 2017).

The present study focused on European populations of *S. woodiana*, though the species has also invaded tropical Asia (1969), Central America (1982), the USA (2010), and Siberia (2016) (Bespalaya et al., 2017; Watters, 1997). One caveat of our study is that we have not included samples from regions other than from Europe and China. Our analysis, therefore, cannot exclude the possibility of a bridgehead invasion to Europe via another region (van Boheemen et al., 2017). However, we believe that a bridgehead invasion from outside Europe is unlikely. First, invasive populations of *S. woodiana* in south‐eastern Asia (Malaysia, Indonesia, the Philippines) were imported from Taiwan (Watters, 1997) and belong to a different phylogenetic lineage (“tropical invasive lineage A”) (Bolotov et al., 2016), except for the localized, recently discovered population in Myanmar (Vikhrev et al., 2017). Second, a population in a large Siberian river, the Yenisei, has been discovered recently and is limited to a thermally polluted outlet from a power station (Bespalaya et al., 2017). Third, populations from North America were first recorded in 2010 in New Jersey (Bogan et al., 2011), well after the expansion of *S. woodiana* in Europe (Lajtner & Crnčan, 2011). The populations of *S. woodiana* in Central America were likely introduced via trade in the Nile tilapia (*O. niloticus*) from Taiwan (Watters, 1997 and references therein), where the tropical (and not temperate) invasive lineage was recorded. While we believe that historical records make bridgehead invasion to Europe unlikely, we acknowledge that we have no data or tissue samples to refute this possibility and we recognize that their contribution to the European *S. woodiana* invasion remains to be tested.

Invasive species can impinge on native communities in a multitude of ways. The impacts of invasive *S. woodiana* populations on the European biota, including whether they are population‐specific, are not understood. Given its generalist exploitation of fish hosts, *S. woodiana* could potentially decrease the accessibility of fish hosts to native unionid species. Individual fish hosts respond to glochidia parasitism by a partial immunization and cross‐resistance after parasitism by *S. woodiana* glochidia, which has the effect of decreasing recruitment of native European mussels (Donrovich et al., 2017). Unionid mussels are also hosts to a group of parasitic cyprinid fishes, the bitterling (Acheilognathinae). Bitterling oviposits in living unionid mussels, with their embryos developing in the gills of their host mussel over their first weeks of life (Smith, Reichard, Jurajda, & Przybylski, 2004). The European bitterling (*Rhodeus amarus* (Bloch)), a generalist host of all European unionids, shows distinct responses to different *S. woodiana* populations in Europe. While populations from Central Poland were used by *R. amarus* for oviposition, with all bitterling embryos later ejected, *S. woodiana* populations from the southern Czech Republic were completely avoided by *R. amarus*, thereby mitigating the costs of failed development. Hence, the identity of *S. woodiana* populations determines the impact of *S. woodiana* on native *R. amarus*, from almost neutral to highly negative (Reichard, Vrtílek, Douda, & Smith, 2012; Reichard et al., 2015). The current study demonstrated that the two *S. woodiana* populations previously used to test their response to bitterling fish are genetically differentiated (PLLI and CZKY populations, respectively). This finding highlights that different *S. woodiana* populations can have different impacts on native communities, an increasingly recognized feature of many invasive species (Morais & Reichard, 2018).

Testing alternative invasion scenarios in a species that readily overcomes natural dispersal barriers is challenging (Estoup & Guillemaud, 2010). Here, we used a novel exploratory approach by testing all pairwise associations between potentially linked populations using an ABC modelling framework. This approach was computationally feasible for our set of 12 potential scenarios across 11 highly admixed populations, enabling us to test many competing colonization scenarios and to generalize across a multitude of pairwise contrasts, without a priori exclusion of any potential relationship. This method is comparable to the approach of Miller et al. (2005) who compared three introduction scenarios for six populations and shares the rationale with the stepwise procedure that requires straightforward hypotheses about invasion pathways based on historical data and/or pronounced genetic structure (e.g., Konečný et al., 2013; Lombaert et al., 2014) and a tournament approach (Stone et al., 2017). Ultimately, it proved useful in providing insights into the origin of European populations of *S. woodiana* from complex evolutionary scenarios and a large sample of sampled populations.

Our understanding of biological invasions has been greatly facilitated through testing ecological and evolutionary invasion scenarios using population genetic data. Identifying the source, timing, and process of an invasion contributes to our capacity to respond to the ongoing spread of non‐native species, thereby improving our ability to predict the invasiveness potential of particular species, lineages, and genotypes (Lockwood et al., 2013). We combined mitochondrial and nuclear markers to demonstrate that *S. woodiana*, a highly invasive species of freshwater mussel, has likely colonized Europe from a single source region. There is no evidence of the recent arrival of a novel genotype. A recently evolved cold tolerance that hypothetically triggered the ongoing invasion of *S. woodiana* in Europe likely arose through in situ adaptation. The current European populations of *S. woodiana* are linked through a network of admixture events between the original source populations and derived ones. Their fine‐scale genetic distinctiveness is likely related to serial founder effects. It remains to be fully investigated how and why different populations of *S. woodiana* vary in their invasive potential and their population‐specific impacts on native taxa and communities. Our results also indicate that adaptations of non‐native species to a new environment may include a capacity for in situ evolution of cold tolerance, with grave implications for their invasiveness.

## AUTHORS’ CONTRIBUTIONS

The study was conceived by M.R. Material was collected by O.P., L.P., M.R., K.D., and C.S. Genotyping was carried by O.P., with the help of V.B., L.P., and J.B. Mitochondrial data were analyzed by O.P. Microsatellite data (including the ABC analysis) were analyzed by A.K., with the help from O.P. The manuscript was drafted by A.K., O.P., and M.R., with significant contribution from all authors. We thank Jasna Lajtner, Teodora Trichkova, Zdravko Hubenov, Marianna Soroka, Nicoletta Riccardi, Irene Guarneri, Vincent Prié, Huanzhang Liu, and Wenjing Yi for providing samples and two anonymous referees for constructive comments that improved the ms.

## DATA ARCHIVING STATEMENT

DNA sequences have been deposited in GenBank: Accession Numbers MG515731‐MG515742. Microsatellite data have been deposited in Figshare: https://doi.org/10.6084/m9.figshare.6895682.
