# Supplementary material for: Modelling the invasion history of Sinanodonta woodiana in Europe: Tracking the routes of a sedentary aquatic invader with mobile parasitic larvae
Source: Evol Appl. 2018 Oct 20;11(10):1975–89. doi: 10.1111/eva.12700 (PMC6231479; doi:10.1111/eva.12700)

**Supplemental Information for:**

**Modelling the invasion history of *Sinanodonta woodiana* in Europe: tracking the routes of a sedentary aquatic invader with mobile parasitic larvae**

**Table of Contents:**

**File S1 Page 2**

**Table S1 Page 3**

**Table S2 Page 8**

**Table S3 Page 9**

**Table S4 Page 10**

**Table S5 Page 14**

**Table S6 Page 15**

**Figure S1 Page 16**

**Figure S2 Page 17**

**Figure S3 Page 21**

**Figure S4 Page 22**

**Figure S5 Page 25**

**Figure S6 Page 26**

**File S1.** **Genotyping details**

Genomic DNA was extracted from 500 tissue samples with the NucleoSpin® Tissue kit (MachereyeNagel GmbH & Co. KG, Düren, Germany), according to the manufacturer’s instructions. We amplified a 370 bp fragment of the mitochondrial gene for cytochrome oxidase c subunit I (COI) using the primers LCO1490 and HCO2198 as described by [Folmer et al., (1994](#_ENREF_3)).

The amplification was performed in 25 μl of a solution containing 10 μM Tris–HCl (pH 8.8 at 25°C), 50 μM KCl, 0.08% (v/v) Nonidet P40, 2.5 μM MgCl_2_, each dNTP at 0.1 μM, each primer at 0.1 μM, 1 unit of Taq DNA polymerase (Fermentas UAB, Vilnius, Lithuania), and approximately 10 ng of DNA template. The temperature profile of the polymerase chain reaction for the COI marker consisted of initial denaturation at 94°C for 30 s, followed by 5 cycles at 93°C for 30 s, 45°C for 45 s, 72°C for 45 s, followed by 30 cycles at 93°C for 30 s, 55°C for 45 s, 72°C for 45 s, and a final extension step performed at 72°C for 10 min. The PCR products were separated on 1% agarose gel, and the fragments of interest purified using the GeneJET™ Gel Extraction Kit (Fermentas UAB, Vilnius, Lithuania). Unidirectional sequencing was performed by Macrogen Europe. Sequences were aligned using CodonCode Aligner 3.7.1. (CodonCode Corporation, Dedham, MA, USA) and verified manually.

We genotyped 17 microsatellite loci developed specifically for *S. woodiana* ([Popa et al., 2015](#_ENREF_6)). PCR amplifications were conducted with the Qiagen Multiplex PCR kit (QiagenTM) in a 10 ml reaction volume containing 5 ml of Multiplex PCR Master Mix (2X) (including HotStarTaq Plus DNA Polymerase, Multiplex PCR Buffer with 3 mM MgCl_2_), between 0.05 and 0.4 mM primer ([Popa et al., 2015](#_ENREF_6)), 1 ml genomic DNA (10 ng) and RNase-free water. A touchdown PCR was performed with the following thermocycling regime: 95^º^C (15 min) followed by 5 cycles of 94^º^C (30 s); annealing temperature stepdowns every 1 cycle of 1ºC (from 60ºC to 55ºC for sets 1 and 2, from 64ºC to 59ºC for set 3, 90 s); 72ºC (90 s). The annealing temperature for the final 30 cycles was 55ºC (59ºC for set 3), with denaturation and extension phases as above. PCR products were sized by capillary electrophoresis using an ABI Prism® 3130 Genetic Analyzer (Applied Biosystems, Foster City, USA) and the GeneScan™ 500 LIZ® Size Standard (Applied Biosystems, Foster City, USA). Alleles were scored in GENEMAPPER v. 5.0 (Applied Biosystems, Foster City, USA) and double-checked manually. The presence of null alleles for each locus and population was assessed with FreeNA (Chapuis & Estoup, 2007).

**Table S1.** List of haplotypes of *S. woodiana* recovered from GenBank and genotyped during the current study. Our haplotype code (corresponding to Fig. 2) and haplotype codes *sensu* Bolotov et al., (2016) and Vikhrev et al., (2017) are listed.

| GenBank Access. No. | Haplotype | Locality | Reference | Bolotov and Vikhrev |
| --- | --- | --- | --- | --- |
| GQ451867 | h6 | South Korea | Direct submission | hapG2 |
| GQ451868 | h7 | South Korea | Direct submission | hapG1 |
| JQ253893 | h2 | Ukraine | Direct submission | hapE3 |
| JQ253894 | h2 | Ukraine | Direct submission | hapE3 |
| AB055627 | h1 | Japan | Direct submission | hapC4 |
| KJ434482 | h2 | China, Jiangxi Poyang | Direct submission | hapE3 |
| KJ434483 | h2 | China, Jiangxi Poyang | Direct submission | hapE2 |
| KJ434484 | h2 | China, Jiangxi Poyang | Direct submission | hapE3 |
| KJ434485 | h2 | China, Jiangxi Poyang | Direct submission | hapE3 |
| KJ434486 | h8 | China, Jiangxi Poyang | Direct submission | hapE1 |
| KJ434487 | h9 | China, Jiangxi Poyang | Direct submission | hapB1 |
| KJ434488 | h10 | China, Jiangxi Poyang | Direct submission | hapD2 |
| KJ434489 | h10 | China, Jiangxi Poyang | Direct submission | hapD1 |
| KJ434490 | h10 | China, Jiangxi Poyang | Direct submission | hapD1 |
| HQ283344 | h2 | Poland | Direct submission | hapE3 |
| HQ283345 | h2 | Poland | Direct submission | hapE3 |
| HQ283346 | h2 | Poland | Direct submission | hapE3 |
| HQ283347 | h2 | Poland | Direct submission | hapE3 |
| HQ283348 | h2 | Poland | Direct submission | hapE3 |
| AF468683 | h2 | Poland | ([Soroka, 2005](#_ENREF_5)) | hapE3 |
| EF440349 | h2 | Poland | ([Soroka, 2010](#_ENREF_6)) | hapE3 |
| KJ125078 | h2 | Poland | ([Soroka et al., 2014](#_ENREF_7)) | hapE3 |
| KJ125079 | h2 | Hungary | ([Soroka et al., 2014](#_ENREF_7)) | hapE3 |
| KF731775 | h2 | Italy, Lake Maggiore | ([Guarneri et al., 2014](#_ENREF_3)) | hapE3 |
| KF731776 | h2 | Italy, River Po | ([Guarneri et al., 2014](#_ENREF_3)) | hapE3 |
| KF731777 | h2 | Italy, River Po | ([Guarneri et al., 2014](#_ENREF_3)) | hapE3 |
| KM272949 | h11 | China | (Zhang et al., 2016) | hapE4 |
| KU891641 | h12 | Indonesia: West Flores, Wae Racang River | ([Bolotov et al., 2016](#_ENREF_1)) | hapA8 |
| KU891642 | h12 | Indonesia: West Flores, Wae Racang River | ([Bolotov et al., 2016](#_ENREF_1)) | hapA8 |
| KX051315 | h13 | Malaysia: Selangor, abandoned mining pool | ([Zieritz et al., 2016](#_ENREF_8)) | hapA5 |
| KX051316 | h13 | Malaysia: Kelantan, Sg. Semerak | ([Zieritz et al., 2016](#_ENREF_8)) | hapA3 |
| KX051317 | h13 | Malaysia: Negeri Sembilan, fish pond | ([Zieritz et al., 2016](#_ENREF_8)) | hapA4 |
| KX051318 | h12 | Malaysia: Malacca, Chohong, Kesang, Kampung Chohong | ([Zieritz et al., 2016](#_ENREF_8)) | hapA8 |
| KX051320 | h12 | Malaysia: Pahang, Lipis, Jelai, Kampung Jeram Besu | ([Zieritz et al., 2016](#_ENREF_8)) | hapA8 |
| KX051321 | h12 | Malaysia: Johor, Chohong, Kesang, Taman Bekoh Jaya | ([Zieritz et al., 2016](#_ENREF_8)) | hapA8 |
| KX051322 | h13 | Malaysia: Kedah, Sg. Pendang | ([Zieritz et al., 2016](#_ENREF_8)) | hapA4 |
| KX051323 | h13 | Malaysia: Perlis | ([Zieritz et al., 2016](#_ENREF_8)) | hapA4 |
| KX051324 | h13 | Malaysia: Selangor, Tasik Semenyih | ([Zieritz et al., 2016](#_ENREF_8)) | hapA3 |
| KX051325 | h13 | Malaysia: Selangor | ([Zieritz et al., 2016](#_ENREF_8)) | hapA2 |
| KX051326 | h12 | Malaysia: Perlis, Tasik Melati | ([Zieritz et al., 2016](#_ENREF_8)) | hapA8 |
| KX051328 | h14 | Malaysia | ([Zieritz et al., 2016](#_ENREF_8)) | hapA1 |
| MF414328 | h2 | Italy | ([Froufe et al., 2017](#_ENREF_2)) |  |
| MF414329 | h2 | Italy | ([Froufe et al., 2017](#_ENREF_2)) |  |
| MF414330 | h2 | Italy | ([Froufe et al., 2017](#_ENREF_2)) |  |
| MF414331 | h2 | Italy | ([Froufe et al., 2017](#_ENREF_2)) |  |
| MF414332 | h2 | Italy | ([Froufe et al., 2017](#_ENREF_2)) |  |
| MF414333 | h2 | Italy | ([Froufe et al., 2017](#_ENREF_2)) |  |
| MF414334 | h2 | Italy | ([Froufe et al., 2017](#_ENREF_2)) |  |
| MF414335 | h2 | Italy | ([Froufe et al., 2017](#_ENREF_2)) |  |
| MF414336 | h2 | Italy | ([Froufe et al., 2017](#_ENREF_2)) |  |
| MF414337 | h2 | Italy | ([Froufe et al., 2017](#_ENREF_2)) |  |
| MF414338 | h2 | Italy | ([Froufe et al., 2017](#_ENREF_2)) |  |
| MF414339 | h2 | Italy | ([Froufe et al., 2017](#_ENREF_2)) |  |
| MF414340 | h2 | Italy | ([Froufe et al., 2017](#_ENREF_2)) |  |
| MF414341 | h2 | Italy | ([Froufe et al., 2017](#_ENREF_2)) |  |
| MF414342 | h2 | Italy | ([Froufe et al., 2017](#_ENREF_2)) |  |
| MF414343 | h2 | Italy | ([Froufe et al., 2017](#_ENREF_2)) |  |
| MF414344 | h2 | Italy | ([Froufe et al., 2017](#_ENREF_2)) |  |
| MF414345 | h2 | Italy | ([Froufe et al., 2017](#_ENREF_2)) |  |
| MF414346 | h2 | Italy | ([Froufe et al., 2017](#_ENREF_2)) |  |
| MF414347 | h2 | Italy | ([Froufe et al., 2017](#_ENREF_2)) |  |
| MF414348 | h2 | Italy | ([Froufe et al., 2017](#_ENREF_2)) |  |
| MF414349 | h2 | Italy | ([Froufe et al., 2017](#_ENREF_2)) |  |
| MF414350 | h2 | Italy | ([Froufe et al., 2017](#_ENREF_2)) |  |
| MF414351 | h2 | Italy | ([Froufe et al., 2017](#_ENREF_2)) |  |
| MF414352 | h2 | Italy | ([Froufe et al., 2017](#_ENREF_2)) |  |
| MG515731 (BGIS1) | h2 | Bulgaria, Iskar River | Present study |  |
| BGIS 2 | h2 | Bulgaria, Iskar River | Present study |  |
| MG515737 (CZKY_1) | h2 | Czech Republic, Kyjovka River | Present study |  |
| CZKY_18 | h2 | Czech Republic, Kyjovka River | Present study |  |
| CZKY_19 | h2 | Czech Republic, Kyjovka River | Present study |  |
| CZKY_2 | h2 | Czech Republic, Kyjovka River | Present study |  |
| CZKY_26 | h2 | Czech Republic, Kyjovka River | Present study |  |
| CZKY_27 | h2 | Czech Republic, Kyjovka River | Present study |  |
| CZKY_28 | h2 | Czech Republic, Kyjovka River | Present study |  |
| CZKY_29 | h2 | Czech Republic, Kyjovka River | Present study |  |
| CZKY_30 | h2 | Czech Republic, Kyjovka River | Present study |  |
| CZKY_31 | h2 | Czech Republic, Kyjovka River | Present study |  |
| CZKY_8 | h2 | Czech Republic, Kyjovka River | Present study |  |
| MG515738 (CZTR_2) | h2 | Czech Republic, Trebonsko fishpond | Present study |  |
| CZTR_3 | h2 | Czech Republic, Trebonsko fishpond | Present study |  |
| CZTR_8 | h2 | Czech Republic, Trebonsko fishpond | Present study |  |
| CZTR_9 | h2 | Czech Republic, Trebonsko fishpond | Present study |  |
| MG515740 (PLKO_19) | h2 | Poland, Konin Lake | Present study |  |
| PLKO_20 | h2 | Poland, Konin Lake | Present study |  |
| PLKO_26 | h2 | Poland, Konin Lake | Present study |  |
| PLKO_3 | h2 | Poland, Konin Lake | Present study |  |
| PLKO_9 | h2 | Poland, Konin Lake | Present study |  |
| MG515741 (ROSV_21) | h2 | Romania, Svinița, Danube River | Present study |  |
| ROSV_22 | h2 | Romania, Svinița, Danube River | Present study |  |
| ROSV_9 | h2 | Romania, Svinița, Danube River | Present study |  |
| MG515742 (ROVA_11) | h2 | Romania, Vădeni, Prut River | Present study |  |
| ROVA_12 | h2 | Romania, Vădeni, Prut River | Present study |  |
| MG515739 (FR_1) | h2 | France, near Arles | Present study |  |
| FR_2 | h2 | France, near Arles | Present study |  |
| ITMA_1 | h2 | Italy, Maggiore Lake | Present study |  |
| ITMA_2 | h2 | Italy, Maggiore Lake | Present study |  |
| ITMA_3 | h2 | Italy, Maggiore Lake | Present study |  |
| ITMA_4 | h2 | Italy, Maggiore Lake | Present study |  |
| ITMA_5 | h2 | Italy, Maggiore Lake | Present study |  |
| ITPO_1 | h2 | Italy, Po River | Present study |  |
| ITPO_2 | h2 | Italy, Po River | Present study |  |
| ITPO_3 | h2 | Italy, Po River | Present study |  |
| ITPO_6 | h2 | Italy, Po River | Present study |  |
| ITPO_9 | h2 | Italy, Po River | Present study |  |
| MG515732 (CNBA_25) | h3 | China, Baoan Lake | Present study |  |
| MG515733 (CNBA_26) | h4 | China, Baoan Lake | Present study |  |
| MG515734 (CNBA_27) | h2 | China, Baoan Lake | Present study |  |
| MG515735 (CNNA_1) | h2 | China, Nanchang | Present study |  |
| CNNA_10 | h2 | China, Nanchang | Present study |  |
| CNNA_11 | h2 | China, Nanchang | Present study |  |
| CNNA_12 | h2 | China, Nanchang | Present study |  |
| CNNA_13 | h2 | China, Nanchang | Present study |  |
| MG515736 (CNNA_14) | h5 | China, Nanchang | Present study |  |
| CNNA_15 | h2 | China, Nanchang | Present study |  |
| CNNA_16 | h2 | China, Nanchang | Present study |  |
| CNNA_17 | h2 | China, Nanchang | Present study |  |
| CNNA_18 | h2 | China, Nanchang | Present study |  |
| CNNA_19 | h2 | China, Nanchang | Present study |  |
| CNNA_20 | h2 | China, Nanchang | Present study |  |
| CNNA_21 | h5 | China, Nanchang | Present study |  |
| CNNA_22 | h2 | China, Nanchang | Present study |  |
| CNNA_23 | h2 | China, Nanchang | Present study |  |
| CNNA_24 | h2 | China, Nanchang | Present study |  |
| CNNA_25 | h2 | China, Nanchang | Present study |  |
| CNNA_26 | h2 | China, Nanchang | Present study |  |
| CNNA_9 | h5 | China, Nanchang | Present study |  |

**Table S2** Summary of pairwise ABC comparisons between 11 *Sinanodonta woodiana* populations (using a uniform prior distribution of parameters) with the most supported evolutionary scenarios for each of 55 analyses and their explanation.

The selected scenarios (SC) in "single-winner" analyses (orange) had the highest relative posterior probability with not overlapping 95% Credible Intervals (CIs). In the case of two selected scenarios (with overlapping CIs; i.e. "double-winner" analyses, yellow), they are presented in order of decreasing posterior probability. Comparisons with three or more similarly supported scenarios are referred to as "many" (no colour highlight). The source population for both the first (F, in rows) or the second (S, in columns) populations is provided. ADM indicates admixture, A indicates an anticipated ancestral population, and U denotes an unsampled population. For population sample codes see Table 1. Bold text highlights the comparisons and relationships that are informative for interpretation of *S. woodiana* invasion history, discussed in the main text and demonstrated in Fig. 4 and S5

| S pop | BGIS | | | ROSV | | | ROMU | | | CZKY | | | CZTR | | | PLSZ | | | PLKO | | | PLLI | | | PLOP | | | PLSP | | |
| --- | --- | --- | --- | --- | --- | --- | --- | --- | --- | --- | --- | --- | --- | --- | --- | --- | --- | --- | --- | --- | --- | --- | --- | --- | --- | --- | --- | --- | --- | --- |
|  | SC | Source of | | SC | Source of | | SC | Source of | | SC | Source of | | SC | Source of | | SC | Source of | | SC | Source of | | SC | Source of | | SC | Source of | | SC | Source of | |
| F pop |  | F | S |  | F | S |  | F | S |  | F | S |  | F | S |  | F | S |  | F | S |  | F | S |  | F | S |  | F | S |
| ROSV | 12, 11 | U, ADM: BGIS+U | ADM: ROSV+U, U |  |  |  |  |  |  |  |  |  |  |  |  |  |  |  |  |  |  |  |  |  |  |  |  |  |  |  |
| ROMU | **7** | **A** | **ADM: ROMU+U** | many |  |  |  |  |  |  |  |  |  |  |  |  |  |  |  |  |  |  |  |  |  |  |  |  |  |  |
| CZKY | 6 | U | U | **8** | **ADM: ROSV+U** | **A** | **8** | **ADM: ROMU+U** | **A** |  |  |  |  |  |  |  |  |  |  |  |  |  |  |  |  |  |  |  |  |  |
| CZTR | 6 | U | U | 5, 6 | U, U | A, U | **8** | **ADM: ROMU+U** | **A** | 11, 6 | ADM: CZKY+U, U | U, U |  |  |  |  |  |  |  |  |  |  |  |  |  |  |  |  |  |  |
| PLSZ | 6, 12 | U, U | U, ADM: PLSZ+U | 6 | U | U | many |  |  | 6, 4 | U, A | U, U | 6, 4 | U, A | U, U |  |  |  |  |  |  |  |  |  |  |  |  |  |  |  |
| PLKO | **12** | U | **ADM: PLKO+U** | **12** | U | **ADM: PLKO+U** | **8** | **ADM: ROMU+U** | **A** | **4** | **A** | U | 6 | U | U | many |  |  |  |  |  |  |  |  |  |  |  |  |  |  |
| PLLI | many |  |  | **11** | **ADM: ROSV+U** | U | **11, 8** | **ADM: ROMU+U, ADM: ROMU+U** | U, A | 11, 6 | ADM: CZKY+U, U | U, U | 6 | U | U | 6 | U | U | **11** | **ADM: PLKO+U** | U |  |  |  |  |  |  |  |  |  |
| PLOP | 6 | U | U | **8** | **ADM: ROSV+U** | **A** | **8** | **ADM: ROMU+U** | **A** | 6 | U | U | 6 | U | U | 5, 6 | U, U | A, U | 6 | U | U | **12** | U | **ADM: PLOP+U** |  |  |  |  |  |  |
| PLSP | 6 | U | U | **12** | U | **ADM: PLSP+U** | many |  |  | 11, 6 | ADM: CZKY+U, U | U, U | many |  |  | many |  |  | 6 | U | U | 6 | U | U | 6, 11 | U, ADM: PLOP+U | U, U |  |  |  |
| FR | many |  |  | 11, 12 | ADM: ROSV+U, U | U, ADM: FR+U | 11, 12 | ADM: ROMU+U, U | U, ADM: FR+U | **7** | **A** | **ADM: FR+U** | many |  |  | **7, 2** | **A, A** | **ADM: FR+U, FR** | **7** | **A** | **ADM: FR+U** | many |  |  | **7** | **A** | **ADM: FR+U** | **7** | **A** | **ADM: FR+U** |

**Table S3** Population genetic differentiation expressed by pairwise *F*_ST_ values (below the diagonal) and mean *F*_ST_ for each sampling site calculated with FSTAT version 2.9.3 (Goudet, 2001). P-values for each pairwise *F*_ST_ are indicated above the diagonal (values in bold are significant at the 0.05 level after correction for multiple comparisons). For population sample codes see Table 1.

(a) non-native range (Europe)

| Site | BGDA | BGIS | ROSV | ROMU | HR | HUDA | HUBA | CZKY | CZTR | PLSZ | PLKO | PLLI | PLOP | PLSP | ITPO | FR | Mean F_ST_ |
| --- | --- | --- | --- | --- | --- | --- | --- | --- | --- | --- | --- | --- | --- | --- | --- | --- | --- |
| BGDA |  | 0.00853 | **0.00001** | 0.00122 | **0.00018** | **0.00013** | **0.00002** | **0.00001** | 0.00106 | **0.00001** | **0.00001** | **0.00001** | **0.00001** | **0.00001** | **0.00001** | 0.08291 | 0.033 |
| BGIS | 0.011 |  | **0.00001** | 0.00180 | **0.00001** | **0.00022** | **0.00003** | **0.00001** | **0.00013** | **0.00001** | **0.00001** | **0.00001** | **0.00001** | **0.00001** | **0.00002** | 0.22239 | 0.042 |
| ROSV | 0.034 | 0.032 |  | **0.00004** | **0.00002** | 0.02493 | **0.00001** | **0.00001** | **0.00002** | **0.00001** | **0.00001** | **0.00001** | **0.00001** | **0.00001** | **0.00003** | 0.11791 | 0.042 |
| ROMU | 0.029 | 0.026 | 0.035 |  | 0.00412 | 0.05490 | **0.00003** | **0.00001** | 0.01046 | **0.00002** | **0.00023** | **0.00001** | **0.00001** | **0.00001** | **0.00001** | 0.13416 | 0.058 |
| HR | 0.026 | 0.026 | 0.023 | 0.032 |  | 0.09286 | **0.00001** | **0.00001** | 0.00762 | **0.00001** | **0.00001** | **0.00001** | **0.00001** | **0.00001** | **0.00001** | 0.03386 | 0.044 |
| HUDA | 0.035 | 0.043 | 0.016 | 0.047 | 0.016 |  | 0.00153 | **0.00001** | **0.00015** | **0.00007** | 0.00168 | **0.00003** | **0.00001** | **0.00001** | **0.00016** | 0.10922 | 0.046 |
| HUBA | 0.032 | 0.029 | 0.044 | 0.065 | 0.035 | 0.035 |  | **0.00001** | 0.03772 | **0.00001** | **0.00001** | **0.00001** | **0.00001** | **0.00001** | **0.00001** | 0.09458 | 0.046 |
| CZKY | 0.040 | 0.073 | 0.057 | 0.096 | 0.059 | 0.063 | 0.053 |  | **0.00001** | **0.00001** | **0.00001** | **0.00001** | **0.00001** | **0.00001** | **0.00001** | **0.00001** | 0.060 |
| CZTR | 0.023 | 0.034 | 0.038 | 0.043 | 0.021 | 0.044 | 0.013 | 0.048 |  | **0.00001** | **0.00001** | **0.00001** | **0.00001** | **0.00001** | **0.00002** | 0.05237 | 0.046 |
| PLSZ | 0.034 | 0.044 | 0.065 | 0.081 | 0.059 | 0.062 | 0.066 | 0.078 | 0.077 |  | **0.00001** | **0.00001** | **0.00001** | **0.00001** | **0.00001** | 0.10952 | 0.063 |
| PLKO | 0.037 | 0.040 | 0.042 | 0.038 | 0.066 | 0.046 | 0.059 | 0.085 | 0.077 | 0.058 |  | **0.00001** | **0.00001** | **0.00001** | **0.00001** | 0.00104 | 0.059 |
| PLLI | 0.022 | 0.027 | 0.039 | 0.052 | 0.050 | 0.047 | 0.019 | 0.049 | 0.038 | 0.038 | 0.022 |  | **0.00001** | **0.00001** | **0.00001** | 0.06213 | 0.041 |
| PLOP | 0.031 | 0.042 | 0.045 | 0.063 | 0.054 | 0.054 | 0.048 | 0.049 | 0.055 | 0.051 | 0.060 | 0.037 |  | **0.00001** | **0.00008** | 0.00373 | 0.050 |
| PLSP | 0.072 | 0.117 | 0.089 | 0.144 | 0.081 | 0.087 | 0.084 | 0.057 | 0.068 | 0.138 | 0.133 | 0.097 | 0.101 |  | **0.00001** | **0.00001** | 0.101 |
| ITPO | 0.058 | 0.074 | 0.057 | 0.096 | 0.082 | 0.071 | 0.092 | 0.043 | 0.076 | 0.081 | 0.094 | 0.074 | 0.041 | 0.131 |  | **0.00035** | 0.075 |
| FR | 0.005 | 0.008 | 0.015 | 0.023 | 0.025 | 0.019 | 0.019 | 0.054 | 0.029 | 0.014 | 0.030 | 0.011 | 0.017 | 0.115 | 0.053 |  | 0.029 |

(b) native range (China)

|  | CNSH | CNNA | CNBA | CNHA | CNJI | CNPO | Mean F_ST_ |
| --- | --- | --- | --- | --- | --- | --- | --- |
| CNSH |  | **0.00007** | **0.00007** | **0.00007** | **0.00007** | **0.00007** | 0.100 |
| CNNA | 0.0962 |  | **0.00007** | **0.00007** | **0.00007** | **0.00007** | 0.059 |
| CNBA | 0.0990 | 0.0221 |  | **0.00007** | **0.00007** | **0.00020** | 0.057 |
| CNHA | 0.0587 | 0.0778 | 0.0755 |  | **0.00007** | **0.00007** | 0.080 |
| CNJI | 0.1637 | 0.0757 | 0.0800 | 0.1204 |  | 0.**00007** | 0.102 |
| CNPO | 0.0869 | 0.0211 | 0.0101 | 0.0661 | 0.0718 |  | 0.051 |

**Table S4** Relative posterior probabilities (with 95% Credible Intervals, CIs) for each of the 55 pairwise ABC comparisons (using a uniform prior distribution of parameters) between 11 *S. woodiana* populations.

Orange-highlighted scenarios indicate the 31 comparisons that resulted in a single supported scenario with the highest posterior probability and not overlapping CIs. Yellow-highlighted scenarios indicate cases when more than one well-supported scenario per comparison had overlapping CIs. For population sample codes see Table 1.

|  | **ROSV vs BGIS** | | **ROMU vs BGIS** | | **CZKY vs BGIS** | | **CZTR vs BGIS** | | **PLSZ vs BGIS** | | **PLKO vs BGIS** | | **PLLI vs BGIS** | | **PLOP vs BGIS** | | **PLSP vs BGIS** | |
| --- | --- | --- | --- | --- | --- | --- | --- | --- | --- | --- | --- | --- | --- | --- | --- | --- | --- | --- |
| **Sc 1** | 0.0504 | [0.0461,0.0546] | 0.0809 | [0.0749,0.0868] | 0.0801 | [0.0758,0.0844] | 0.092 | [0.0872,0.0968] | 0.0624 | [0.0577,0.0671] | 0.0551 | [0.0495,0.0607] | 0.0513 | [0.0481,0.0545] | 0.0424 | [0.0395,0.0454] | 0.0414 | [0.0366,0.0462] |
| **Sc 2** | 0.0894 | [0.0840,0.0949] | 0.1259 | [0.1180,0.1338] | 0.0434 | [0.0391,0.0476] | 0.0469 | [0.0433,0.0504] | 0.0694 | [0.0634,0.0755] | 0.0607 | [0.0535,0.0680] | 0.0611 | [0.0578,0.0645] | 0.0356 | [0.0326,0.0387] | 0.0564 | [0.0496,0.0631] |
| **Sc 3** | 0.0501 | [0.0457,0.0545] | 0.0209 | [0.0163,0.0255] | 0.049 | [0.0453,0.0528] | 0.0432 | [0.0397,0.0466] | 0.0483 | [0.0436,0.0530] | 0.043 | [0.0376,0.0485] | 0.0613 | [0.0581,0.0646] | 0.0732 | [0.0696,0.0767] | 0.0645 | [0.0593,0.0698] |
| **Sc 4** | 0.0541 | [0.0498,0.0584] | 0.101 | [0.0937,0.1083] | 0.0939 | [0.0894,0.0984] | 0.1034 | [0.0981,0.1088] | 0.0649 | [0.0602,0.0696] | 0.0719 | [0.0654,0.0784] | 0.0444 | [0.0413,0.0476] | 0.0384 | [0.0355,0.0412] | 0.0544 | [0.0496,0.0592] |
| **Sc 5** | 0.0331 | [0.0291,0.0371] | 0.0392 | [0.0346,0.0439] | 0.1199 | [0.1141,0.1257] | 0.1085 | [0.1031,0.1139] | 0.0519 | [0.0477,0.0560] | 0.0634 | [0.0579,0.0690] | 0.0425 | [0.0394,0.0456] | 0.0785 | [0.0750,0.0820] | 0.0769 | [0.0686,0.0851] |
| **Sc 6** | 0.1146 | [0.1084,0.1207] | 0.0948 | [0.0887,0.1009] | 0.1454 | [0.1392,0.1516] | 0.1361 | [0.1303,0.1419] | 0.1462 | [0.1384,0.1540] | 0.1373 | [0.1264,0.1481] | 0.1528 | [0.1473,0.1584] | 0.1786 | [0.1732,0.1840] | 0.1551 | [0.1472,0.1630] |
| **Sc 7** | 0.1064 | [0.1004,0.1124] | 0.1524 | [0.1438,0.1609] | 0.0511 | [0.0465,0.0558] | 0.0553 | [0.0516,0.0591] | 0.0894 | [0.0824,0.0965] | 0.0855 | [0.0768,0.0943] | 0.0706 | [0.0671,0.0741] | 0.029 | [0.0261,0.0319] | 0.0413 | [0.0350,0.0476] |
| **Sc 8** | 0.0559 | [0.0513,0.0604] | 0.0217 | [0.0171,0.0262] | 0.0642 | [0.0602,0.0682] | 0.0487 | [0.0452,0.0522] | 0.0625 | [0.0574,0.0677] | 0.0432 | [0.0379,0.0485] | 0.0696 | [0.0661,0.0730] | 0.0997 | [0.0955,0.1038] | 0.1393 | [0.1314,0.1472] |
| **Sc 9** | 0.0747 | [0.0696,0.0799] | 0.0598 | [0.0548,0.0648] | 0.0814 | [0.0772,0.0857] | 0.0726 | [0.0685,0.0766] | 0.0737 | [0.0684,0.0790] | 0.0419 | [0.0367,0.0472] | 0.0726 | [0.0690,0.0761] | 0.0978 | [0.0937,0.1020] | 0.1139 | [0.1075,0.1204] |
| **Sc 10** | 0.0891 | [0.0836,0.0946] | 0.108 | [0.1009,0.1151] | 0.0896 | [0.0844,0.0948] | 0.0947 | [0.0897,0.0998] | 0.0861 | [0.0801,0.0922] | 0.1226 | [0.1130,0.1323] | 0.0735 | [0.0699,0.0771] | 0.0554 | [0.0522,0.0585] | 0.048 | [0.0428,0.0532] |
| **Sc 11** | 0.1402 | [0.1327,0.1477] | 0.0773 | [0.0719,0.0827] | 0.0791 | [0.0748,0.0834] | 0.0995 | [0.0946,0.1044] | 0.1127 | [0.1054,0.1200] | 0.097 | [0.0900,0.1040] | 0.1447 | [0.1395,0.1500] | 0.162 | [0.1563,0.1677] | 0.1281 | [0.1208,0.1354] |
| **Sc 12** | 0.1421 | [0.1351,0.1490] | 0.1181 | [0.1108,0.1254] | 0.1028 | [0.0972,0.1084] | 0.0991 | [0.0942,0.1040] | 0.1324 | [0.1240,0.1409] | 0.1782 | [0.1659,0.1905] | 0.1555 | [0.1503,0.1606] | 0.1094 | [0.1051,0.1138] | 0.0807 | [0.0747,0.0868] |
|  | **FR vs BGIS** | | **ROMU vs ROSV** | | **CZKY vs ROSV** | | **CZTR vs ROSV** | | **PLSZ vs ROSV** | | **PLKO vs ROSV** | | **PLLI vs ROSV** | | **PLOP vs ROSV** | | **PLSP vs ROSV** | |
| **Sc 1** | 0.0087 | [0.0044,0.0130] | 0.0932 | [0.0861,0.1003] | 0.0497 | [0.0440,0.0554] | 0.1005 | [0.0948,0.1062] | 0.0609 | [0.0561,0.0658] | 0.0367 | [0.0304,0.0431] | 0.0261 | [0.0216,0.0307] | 0.0484 | [0.0435,0.0533] | 0.0367 | [0.0330,0.0404] |
| **Sc 2** | 0.1718 | [0.1656,0.1780] | 0.0854 | [0.0787,0.0920] | 0.0319 | [0.0263,0.0375] | 0.0291 | [0.0250,0.0331] | 0.0596 | [0.0536,0.0657] | 0.0491 | [0.0422,0.0560] | 0.0507 | [0.0458,0.0556] | 0.0156 | [0.0111,0.0201] | 0.0333 | [0.0291,0.0376] |
| **Sc 3** | 0.083 | [0.0786,0.0874] | 0.0474 | [0.0424,0.0523] | 0.1429 | [0.1312,0.1547] | 0.0529 | [0.0488,0.0569] | 0.0556 | [0.0508,0.0603] | 0.0549 | [0.0486,0.0612] | 0.0898 | [0.0841,0.0954] | 0.0974 | [0.0919,0.1029] | 0.0925 | [0.0875,0.0975] |
| **Sc 4** | 0.007 | [0.0026,0.0113] | 0.0874 | [0.0804,0.0944] | 0.0202 | [0.0158,0.0246] | 0.1 | [0.0940,0.1061] | 0.0541 | [0.0497,0.0585] | 0.0368 | [0.0304,0.0431] | 0.0198 | [0.0152,0.0244] | 0.0297 | [0.0251,0.0343] | 0.0317 | [0.0280,0.0353] |
| **Sc 5** | 0.0036 | [0.0000,0.0080] | 0.0645 | [0.0588,0.0701] | 0.0648 | [0.0592,0.0703] | 0.1449 | [0.1375,0.1522] | 0.0686 | [0.0641,0.0731] | 0.0422 | [0.0361,0.0482] | 0.0357 | [0.0313,0.0402] | 0.0928 | [0.0866,0.0990] | 0.0635 | [0.0600,0.0671] |
| **Sc 6** | 0.0763 | [0.0720,0.0806] | 0.1111 | [0.1038,0.1183] | 0.108 | [0.0999,0.1160] | 0.1398 | [0.1329,0.1467] | 0.1405 | [0.1331,0.1479] | 0.1292 | [0.1175,0.1408] | 0.1598 | [0.1512,0.1684] | 0.1044 | [0.0988,0.1100] | 0.132 | [0.1254,0.1385] |
| **Sc 7** | 0.1706 | [0.1641,0.1771] | 0.0877 | [0.0810,0.0944] | 0.0248 | [0.0200,0.0296] | 0.03 | [0.0259,0.0341] | 0.066 | [0.0597,0.0722] | 0.0751 | [0.0673,0.0829] | 0.045 | [0.0403,0.0497] | 0.0197 | [0.0153,0.0242] | 0.0455 | [0.0409,0.0501] |
| **Sc 8** | 0.0675 | [0.0634,0.0717] | 0.0427 | [0.0379,0.0474] | 0.2641 | [0.2504,0.2779] | 0.0567 | [0.0527,0.0606] | 0.1004 | [0.0937,0.1072] | 0.0568 | [0.0507,0.0629] | 0.126 | [0.1193,0.1327] | 0.168 | [0.1599,0.1762] | 0.1253 | [0.1195,0.1311] |
| **Sc 9** | 0.052 | [0.0478,0.0561] | 0.0922 | [0.0852,0.0993] | 0.0496 | [0.0448,0.0544] | 0.0856 | [0.0807,0.0905] | 0.0813 | [0.0758,0.0868] | 0.0402 | [0.0341,0.0464] | 0.0766 | [0.0714,0.0819] | 0.1108 | [0.1041,0.1175] | 0.0713 | [0.0672,0.0753] |
| **Sc 10** | 0.0421 | [0.0381,0.0461] | 0.0984 | [0.0911,0.1057] | 0.0262 | [0.0217,0.0307] | 0.095 | [0.0891,0.1009] | 0.0831 | [0.0769,0.0893] | 0.1801 | [0.1677,0.1924] | 0.0433 | [0.0387,0.0479] | 0.0717 | [0.0664,0.0770] | 0.0919 | [0.0867,0.0970] |
| **Sc 11** | 0.1574 | [0.1514,0.1635] | 0.0872 | [0.0810,0.0933] | 0.1083 | [0.1004,0.1163] | 0.0862 | [0.0813,0.0911] | 0.108 | [0.1013,0.1146] | 0.0941 | [0.0875,0.1006] | 0.2001 | [0.1910,0.2091] | 0.1416 | [0.1337,0.1494] | 0.1137 | [0.1086,0.1189] |
| **Sc 12** | 0.16 | [0.1539,0.1661] | 0.1029 | [0.0958,0.1100] | 0.1095 | [0.1011,0.1179] | 0.0793 | [0.0743,0.0842] | 0.122 | [0.1141,0.1299] | 0.205 | [0.1928,0.2171] | 0.127 | [0.1207,0.1334] | 0.1 | [0.0945,0.1055] | 0.1627 | [0.1552,0.1702] |

|  | **FR vs ROSV** | | **CZKY vs ROMU** | | **CZTR vs ROMU** | | **PLSZ vs ROMU** | | **PLKO vs ROMU** | | **PLLI vs ROMU** | | **PLOP vs ROMU** | | **PLSP vs ROMU** | | **FR vs ROMU** | |
| --- | --- | --- | --- | --- | --- | --- | --- | --- | --- | --- | --- | --- | --- | --- | --- | --- | --- | --- |
| **Sc 1** | 0.0267 | [0.0237,0.0298] | 0.0578 | [0.0530,0.0626] | 0.0369 | [0.0319,0.0418] | 0.0944 | [0.0878,0.1009] | 0.0554 | [0.0517,0.0591] | 0.0297 | [0.0258,0.0337] | 0.0436 | [0.0396,0.0477] | 0.0553 | [0.0510,0.0596] | 0.0539 | [0.0518,0.0560] |
| **Sc 2** | 0.1277 | [0.1233,0.1321] | 0.0334 | [0.0280,0.0387] | 0.0364 | [0.0316,0.0411] | 0.0385 | [0.0332,0.0438] | 0.0262 | [0.0231,0.0293] | 0.0378 | [0.0337,0.0418] | 0.0288 | [0.0245,0.0332] | 0.0274 | [0.0225,0.0323] | 0.0997 | [0.0970,0.1023] |
| **Sc 3** | 0.0886 | [0.0850,0.0922] | 0.0906 | [0.0846,0.0966] | 0.1397 | [0.1320,0.1473] | 0.0615 | [0.0562,0.0667] | 0.1205 | [0.1154,0.1256] | 0.1283 | [0.1221,0.1346] | 0.1157 | [0.1096,0.1218] | 0.0679 | [0.0630,0.0728] | 0.0928 | [0.0902,0.0953] |
| **Sc 4** | 0.0136 | [0.0105,0.0166] | 0.0511 | [0.0465,0.0558] | 0.0161 | [0.0111,0.0212] | 0.084 | [0.0782,0.0898] | 0.0344 | [0.0310,0.0377] | 0.0188 | [0.0148,0.0227] | 0.032 | [0.0280,0.0360] | 0.061 | [0.0567,0.0653] | 0.038 | [0.0359,0.0400] |
| **Sc 5** | 0.0135 | [0.0104,0.0166] | 0.109 | [0.1006,0.1175] | 0.0479 | [0.0428,0.0530] | 0.1201 | [0.1134,0.1268] | 0.0673 | [0.0634,0.0712] | 0.0436 | [0.0397,0.0476] | 0.0883 | [0.0835,0.0931] | 0.1229 | [0.1171,0.1288] | 0.0357 | [0.0336,0.0377] |
| **Sc 6** | 0.0908 | [0.0871,0.0945] | 0.1178 | [0.1116,0.1239] | 0.1057 | [0.0996,0.1118] | 0.1144 | [0.1076,0.1212] | 0.0943 | [0.0900,0.0986] | 0.131 | [0.1251,0.1368] | 0.1233 | [0.1172,0.1294] | 0.1314 | [0.1248,0.1379] | 0.0865 | [0.0841,0.0890] |
| **Sc 7** | 0.1244 | [0.1201,0.1287] | 0.0262 | [0.0211,0.0314] | 0.0406 | [0.0359,0.0454] | 0.0343 | [0.0293,0.0393] | 0.0287 | [0.0255,0.0318] | 0.0395 | [0.0355,0.0436] | 0.0274 | [0.0231,0.0318] | 0.0235 | [0.0185,0.0284] | 0.1026 | [0.0999,0.1053] |
| **Sc 8** | 0.0956 | [0.0918,0.0994] | 0.175 | [0.1658,0.1841] | 0.1771 | [0.1678,0.1864] | 0.1035 | [0.0965,0.1105] | 0.1577 | [0.1520,0.1635] | 0.1489 | [0.1421,0.1558] | 0.1403 | [0.1339,0.1467] | 0.1237 | [0.1168,0.1305] | 0.0901 | [0.0876,0.0926] |
| **Sc 9** | 0.0715 | [0.0680,0.0750] | 0.1247 | [0.1179,0.1315] | 0.0808 | [0.0752,0.0865] | 0.1187 | [0.1111,0.1263] | 0.0957 | [0.0912,0.1003] | 0.076 | [0.0714,0.0805] | 0.0797 | [0.0750,0.0844] | 0.1274 | [0.1207,0.1340] | 0.0813 | [0.0789,0.0837] |
| **Sc 10** | 0.0661 | [0.0628,0.0695] | 0.0403 | [0.0356,0.0450] | 0.0629 | [0.0578,0.0681] | 0.0863 | [0.0797,0.0930] | 0.0919 | [0.0872,0.0966] | 0.0524 | [0.0483,0.0566] | 0.0753 | [0.0701,0.0805] | 0.0627 | [0.0575,0.0679] | 0.0835 | [0.0810,0.0859] |
| **Sc 11** | 0.1444 | [0.1396,0.1491] | 0.0964 | [0.0906,0.1022] | 0.1424 | [0.1335,0.1512] | 0.0714 | [0.0662,0.0766] | 0.1118 | [0.1071,0.1165] | 0.1582 | [0.1517,0.1647] | 0.1239 | [0.1178,0.1300] | 0.1098 | [0.1035,0.1160] | 0.1186 | [0.1157,0.1216] |
| **Sc 12** | 0.137 | [0.1324,0.1416] | 0.0778 | [0.0721,0.0834] | 0.1135 | [0.1073,0.1198] | 0.0729 | [0.0673,0.0786] | 0.116 | [0.1110,0.1210] | 0.1357 | [0.1296,0.1419] | 0.1215 | [0.1150,0.1281] | 0.0871 | [0.0807,0.0934] | 0.1174 | [0.1146,0.1203] |

|  | **CZTR vs CZKY** | | **PLSZ vs CZKY** | | **PLKO vs CZKY** | | **PLLI vs CZKY** | | **PLOP vs CZKY** | | **PLSP vs CZKY** | | **FR vs CZKY** | | **PLSZ vs CZTR** | | **PLKO vs CZTR** | |
| --- | --- | --- | --- | --- | --- | --- | --- | --- | --- | --- | --- | --- | --- | --- | --- | --- | --- | --- |
| **Sc 1** | 0.0629 | [0.0564,0.0693] | 0.0892 | [0.0828,0.0957] | 0.1087 | [0.1012,0.1162] | 0.0546 | [0.0486,0.0606] | 0.0434 | [0.0389,0.0478] | 0.0631 | [0.0571,0.0691] | 0.0405 | [0.0361,0.0450] | 0.092 | [0.0860,0.0981] | 0.0764 | [0.0712,0.0816] |
| **Sc 2** | 0.0565 | [0.0503,0.0627] | 0.0486 | [0.0438,0.0534] | 0.0362 | [0.0308,0.0415] | 0.0708 | [0.0641,0.0774] | 0.0579 | [0.0533,0.0626] | 0.0326 | [0.0275,0.0377] | 0.117 | [0.1099,0.1242] | 0.0511 | [0.0452,0.0570] | 0.0483 | [0.0424,0.0541] |
| **Sc 3** | 0.0509 | [0.0429,0.0588] | 0.0385 | [0.0332,0.0438] | 0.0257 | [0.0204,0.0310] | 0.0344 | [0.0273,0.0415] | 0.077 | [0.0710,0.0831] | 0.0521 | [0.0470,0.0572] | 0.0269 | [0.0223,0.0314] | 0.034 | [0.0290,0.0391] | 0.0277 | [0.0232,0.0323] |
| **Sc 4** | 0.0795 | [0.0696,0.0895] | 0.1397 | [0.1318,0.1476] | 0.1991 | [0.1897,0.2084] | 0.082 | [0.0720,0.0920] | 0.0383 | [0.0344,0.0423] | 0.0682 | [0.0621,0.0743] | 0.0701 | [0.0650,0.0751] | 0.1382 | [0.1306,0.1459] | 0.1352 | [0.1281,0.1423] |
| **Sc 5** | 0.0733 | [0.0662,0.0803] | 0.0849 | [0.0791,0.0908] | 0.1348 | [0.1267,0.1429] | 0.0419 | [0.0359,0.0479] | 0.0574 | [0.0525,0.0623] | 0.1065 | [0.0991,0.1140] | 0.0257 | [0.0216,0.0298] | 0.1049 | [0.0970,0.1128] | 0.1198 | [0.1113,0.1283] |
| **Sc 6** | 0.1285 | [0.1190,0.1381] | 0.1521 | [0.1442,0.1600] | 0.118 | [0.1106,0.1254] | 0.1387 | [0.1294,0.1481] | 0.1749 | [0.1667,0.1830] | 0.1515 | [0.1429,0.1601] | 0.1095 | [0.1028,0.1162] | 0.1431 | [0.1350,0.1512] | 0.1768 | [0.1680,0.1856] |
| **Sc 7** | 0.1005 | [0.0926,0.1085] | 0.102 | [0.0949,0.1090] | 0.0658 | [0.0592,0.0724] | 0.0979 | [0.0905,0.1052] | 0.077 | [0.0718,0.0822] | 0.0315 | [0.0265,0.0365] | 0.1855 | [0.1765,0.1946] | 0.0459 | [0.0405,0.0514] | 0.0469 | [0.0412,0.0526] |
| **Sc 8** | 0.0509 | [0.0430,0.0588] | 0.0334 | [0.0284,0.0384] | 0.0187 | [0.0138,0.0236] | 0.0661 | [0.0565,0.0756] | 0.1022 | [0.0951,0.1094] | 0.0864 | [0.0802,0.0926] | 0.0343 | [0.0295,0.0392] | 0.0461 | [0.0408,0.0513] | 0.039 | [0.0345,0.0436] |
| **Sc 9** | 0.0947 | [0.0857,0.1036] | 0.053 | [0.0481,0.0580] | 0.0422 | [0.0373,0.0472] | 0.1006 | [0.0913,0.1098] | 0.0554 | [0.0508,0.0601] | 0.1277 | [0.1186,0.1368] | 0.0715 | [0.0657,0.0773] | 0.0929 | [0.0864,0.0994] | 0.0838 | [0.0783,0.0893] |
| **Sc 10** | 0.0703 | [0.0638,0.0767] | 0.1194 | [0.1117,0.1271] | 0.1529 | [0.1426,0.1632] | 0.0606 | [0.0547,0.0665] | 0.0639 | [0.0589,0.0688] | 0.0476 | [0.0424,0.0527] | 0.0929 | [0.0866,0.0992] | 0.076 | [0.0703,0.0817] | 0.0713 | [0.0659,0.0768] |
| **Sc 11** | 0.1327 | [0.1214,0.1439] | 0.0603 | [0.0553,0.0652] | 0.0371 | [0.0322,0.0420] | 0.1487 | [0.1369,0.1606] | 0.1398 | [0.1320,0.1477] | 0.1577 | [0.1482,0.1672] | 0.109 | [0.1020,0.1160] | 0.0783 | [0.0723,0.0842] | 0.0775 | [0.0721,0.0829] |
| **Sc 12** | 0.0994 | [0.0914,0.1075] | 0.0788 | [0.0733,0.0842] | 0.061 | [0.0552,0.0667] | 0.1037 | [0.0960,0.1115] | 0.1127 | [0.1063,0.1191] | 0.0751 | [0.0696,0.0806] | 0.1171 | [0.1101,0.1240] | 0.0975 | [0.0905,0.1045] | 0.0972 | [0.0907,0.1038] |

|  | **PLLI vs CZTR** | | **PLOP vs CZTR** | | **PLSP vs CZTR** | | **FR vs CZTR** | | **PLKO vs PLSZ** | | **PLLI vs PLSZ** | | **PLOP vs PLSZ** | | **PLSP vs PLSZ** | | **FR vs PLSZ** | |
| --- | --- | --- | --- | --- | --- | --- | --- | --- | --- | --- | --- | --- | --- | --- | --- | --- | --- | --- |
| **Sc 1** | 0.0757 | [0.0722,0.0793] | 0.0421 | [0.0385,0.0457] | 0.0372 | [0.0335,0.0408] | 0.0436 | [0.0378,0.0494] | 0.1296 | [0.1193,0.1399] | 0.0642 | [0.0593,0.0691] | 0.0689 | [0.0641,0.0737] | 0.0584 | [0.0530,0.0638] | 0.0267 | [0.0223,0.0312] |
| **Sc 2** | 0.0479 | [0.0448,0.0511] | 0.0337 | [0.0301,0.0373] | 0.0339 | [0.0299,0.0379] | 0.1081 | [0.1009,0.1152] | 0.0358 | [0.0293,0.0422] | 0.0662 | [0.0604,0.0719] | 0.0268 | [0.0228,0.0307] | 0.0347 | [0.0284,0.0411] | 0.1655 | [0.1570,0.1740] |
| **Sc 3** | 0.0423 | [0.0394,0.0451] | 0.0745 | [0.0704,0.0787] | 0.0819 | [0.0775,0.0862] | 0.0498 | [0.0444,0.0552] | 0.0471 | [0.0402,0.0539] | 0.0724 | [0.0659,0.0788] | 0.0805 | [0.0752,0.0857] | 0.0631 | [0.0568,0.0694] | 0.0666 | [0.0617,0.0716] |
| **Sc 4** | 0.1041 | [0.0997,0.1085] | 0.0519 | [0.0480,0.0559] | 0.0354 | [0.0318,0.0390] | 0.0432 | [0.0375,0.0488] | 0.1058 | [0.0977,0.1140] | 0.0505 | [0.0462,0.0548] | 0.0575 | [0.0534,0.0617] | 0.0689 | [0.0636,0.0742] | 0.0188 | [0.0143,0.0233] |
| **Sc 5** | 0.087 | [0.0831,0.0909] | 0.0736 | [0.0695,0.0778] | 0.0718 | [0.0679,0.0757] | 0.026 | [0.0204,0.0315] | 0.1229 | [0.1138,0.1320] | 0.0585 | [0.0538,0.0632] | 0.1524 | [0.1446,0.1601] | 0.1268 | [0.1192,0.1344] | 0.012 | [0.0074,0.0165] |
| **Sc 6** | 0.163 | [0.1576,0.1683] | 0.1769 | [0.1701,0.1838] | 0.1466 | [0.1407,0.1525] | 0.0991 | [0.0918,0.1064] | 0.1182 | [0.1092,0.1271] | 0.1451 | [0.1369,0.1533] | 0.1478 | [0.1411,0.1545] | 0.1417 | [0.1331,0.1503] | 0.0884 | [0.0827,0.0940] |
| **Sc 7** | 0.0528 | [0.0497,0.0560] | 0.0374 | [0.0337,0.0410] | 0.0388 | [0.0346,0.0429] | 0.1326 | [0.1240,0.1412] | 0.0432 | [0.0367,0.0497] | 0.0862 | [0.0795,0.0929] | 0.0244 | [0.0206,0.0282] | 0.0331 | [0.0266,0.0396] | 0.1757 | [0.1676,0.1838] |
| **Sc 8** | 0.0412 | [0.0384,0.0440] | 0.1009 | [0.0960,0.1058] | 0.1294 | [0.1236,0.1351] | 0.0545 | [0.0489,0.0601] | 0.0752 | [0.0669,0.0835] | 0.0725 | [0.0663,0.0787] | 0.0986 | [0.0930,0.1043] | 0.1354 | [0.1253,0.1454] | 0.0654 | [0.0605,0.0704] |
| **Sc 9** | 0.0729 | [0.0694,0.0764] | 0.0842 | [0.0796,0.0888] | 0.0789 | [0.0746,0.0832] | 0.0793 | [0.0723,0.0863] | 0.0967 | [0.0882,0.1052] | 0.0729 | [0.0674,0.0784] | 0.108 | [0.1019,0.1141] | 0.1389 | [0.1300,0.1478] | 0.0626 | [0.0577,0.0676] |
| **Sc 10** | 0.0799 | [0.0761,0.0836] | 0.0593 | [0.0553,0.0633] | 0.0804 | [0.0756,0.0851] | 0.0705 | [0.0644,0.0766] | 0.0863 | [0.0782,0.0945] | 0.0834 | [0.0773,0.0895] | 0.0524 | [0.0483,0.0565] | 0.0595 | [0.0533,0.0658] | 0.0662 | [0.0612,0.0712] |
| **Sc 11** | 0.1117 | [0.1072,0.1163] | 0.1435 | [0.1371,0.1500] | 0.1374 | [0.1314,0.1435] | 0.1509 | [0.1410,0.1607] | 0.0728 | [0.0656,0.0800] | 0.1083 | [0.1009,0.1157] | 0.1183 | [0.1123,0.1243] | 0.093 | [0.0860,0.1000] | 0.1268 | [0.1200,0.1336] |
| **Sc 12** | 0.1215 | [0.1167,0.1263] | 0.1219 | [0.1161,0.1278] | 0.1286 | [0.1225,0.1347] | 0.1424 | [0.1333,0.1515] | 0.0665 | [0.0596,0.0734] | 0.1198 | [0.1118,0.1278] | 0.0645 | [0.0604,0.0685] | 0.0464 | [0.0408,0.0520] | 0.1252 | [0.1186,0.1319] |

|  | **PLLI vs PLKO** | | **PLOP vs PLKO** | | **PLSP vs PLKO** | | **FR vs PLKO** | | **PLOP vs PLLI** | | **PLSP vs PLLI** | | **FR vs PLLI** | | **PLSP vs PLOP** | | **FR vs PLOP** | | **FR vs PLSP** | |
| --- | --- | --- | --- | --- | --- | --- | --- | --- | --- | --- | --- | --- | --- | --- | --- | --- | --- | --- | --- | --- |
| **Sc 1** | 0.0146 | [0.0098,0.0194] | 0.0583 | [0.0547,0.0619] | 0.065 | [0.0613,0.0687] | 0.0351 | [0.0309,0.0394] | 0.019 | [0.0153,0.0226] | 0.0389 | [0.0353,0.0425] | 0.0172 | [0.0129,0.0215] | 0.0245 | [0.0198,0.0293] | 0.0238 | [0.0206,0.0271] | 0.036 | [0.0321,0.0399] |
| **Sc 2** | 0.102 | [0.0945,0.1095] | 0.0359 | [0.0327,0.0391] | 0.0294 | [0.0255,0.0332] | 0.1224 | [0.1161,0.1286] | 0.0443 | [0.0404,0.0482] | 0.0455 | [0.0412,0.0498] | 0.1603 | [0.1535,0.1671] | 0.0551 | [0.0490,0.0611] | 0.1642 | [0.1588,0.1696] | 0.0841 | [0.0798,0.0883] |
| **Sc 3** | 0.0557 | [0.0501,0.0612] | 0.0503 | [0.0469,0.0538] | 0.053 | [0.0490,0.0570] | 0.042 | [0.0376,0.0464] | 0.1195 | [0.1144,0.1245] | 0.0703 | [0.0664,0.0742] | 0.0589 | [0.0545,0.0632] | 0.0636 | [0.0578,0.0694] | 0.0121 | [0.0090,0.0153] | 0.0351 | [0.0307,0.0396] |
| **Sc 4** | 0.0093 | [0.0045,0.0142] | 0.0763 | [0.0725,0.0801] | 0.0835 | [0.0798,0.0873] | 0.0455 | [0.0412,0.0497] | 0.0146 | [0.0109,0.0183] | 0.0485 | [0.0449,0.0521] | 0.017 | [0.0126,0.0214] | 0.0363 | [0.0317,0.0409] | 0.0404 | [0.0371,0.0438] | 0.0783 | [0.0714,0.0852] |
| **Sc 5** | 0.0099 | [0.0050,0.0148] | 0.1217 | [0.1164,0.1269] | 0.1358 | [0.1299,0.1416] | 0.0252 | [0.0210,0.0295] | 0.0266 | [0.0230,0.0301] | 0.083 | [0.0760,0.0900] | 0.0115 | [0.0070,0.0159] | 0.049 | [0.0444,0.0535] | 0.0104 | [0.0071,0.0136] | 0.0313 | [0.0274,0.0352] |
| **Sc 6** | 0.1012 | [0.0936,0.1088] | 0.1756 | [0.1697,0.1814] | 0.1708 | [0.1645,0.1772] | 0.1294 | [0.1228,0.1361] | 0.1473 | [0.1419,0.1526] | 0.1778 | [0.1715,0.1842] | 0.0953 | [0.0902,0.1005] | 0.1835 | [0.1751,0.1919] | 0.0911 | [0.0872,0.0951] | 0.1211 | [0.1162,0.1259] |
| **Sc 7** | 0.1009 | [0.0937,0.1082] | 0.0317 | [0.0287,0.0347] | 0.0225 | [0.0187,0.0262] | 0.1481 | [0.1414,0.1549] | 0.0573 | [0.0533,0.0613] | 0.0423 | [0.0379,0.0466] | 0.1638 | [0.1572,0.1704] | 0.0301 | [0.0251,0.0350] | 0.191 | [0.1855,0.1965] | 0.1657 | [0.1594,0.1720] |
| **Sc 8** | 0.085 | [0.0777,0.0923] | 0.0584 | [0.0549,0.0618] | 0.0802 | [0.0755,0.0848] | 0.0451 | [0.0407,0.0495] | 0.1077 | [0.1032,0.1121] | 0.1029 | [0.0984,0.1074] | 0.0544 | [0.0501,0.0587] | 0.1135 | [0.1056,0.1213] | 0.0219 | [0.0187,0.0251] | 0.028 | [0.0236,0.0325] |
| **Sc 9** | 0.1174 | [0.1069,0.1279] | 0.0907 | [0.0862,0.0951] | 0.1407 | [0.1350,0.1465] | 0.0862 | [0.0806,0.0919] | 0.0403 | [0.0367,0.0438] | 0.0807 | [0.0769,0.0846] | 0.0546 | [0.0502,0.0590] | 0.1456 | [0.1366,0.1545] | 0.0951 | [0.0909,0.0993] | 0.0845 | [0.0798,0.0892] |
| **Sc 10** | 0.044 | [0.0389,0.0491] | 0.0758 | [0.0717,0.0798] | 0.0528 | [0.0491,0.0566] | 0.0654 | [0.0608,0.0700] | 0.0847 | [0.0803,0.0891] | 0.0696 | [0.0655,0.0738] | 0.0555 | [0.0512,0.0598] | 0.0274 | [0.0228,0.0321] | 0.0616 | [0.0581,0.0651] | 0.0951 | [0.0909,0.0994] |
| **Sc 11** | 0.2382 | [0.2255,0.2510] | 0.1315 | [0.1260,0.1370] | 0.0978 | [0.0931,0.1026] | 0.127 | [0.1205,0.1334] | 0.1561 | [0.1499,0.1623] | 0.1305 | [0.1254,0.1355] | 0.1455 | [0.1391,0.1518] | 0.1817 | [0.1717,0.1917] | 0.156 | [0.1507,0.1612] | 0.1238 | [0.1181,0.1296] |
| **Sc 12** | 0.1218 | [0.1137,0.1299] | 0.0939 | [0.0895,0.0982] | 0.0684 | [0.0641,0.0727] | 0.1285 | [0.1222,0.1348] | 0.1828 | [0.1762,0.1894] | 0.11 | [0.1050,0.1151] | 0.1661 | [0.1591,0.1731] | 0.0898 | [0.0837,0.0959] | 0.1322 | [0.1276,0.1369] | 0.1168 | [0.1119,0.1217] |

**Table S5** Comparison of scenario choice in the ABC analyses using two different sets of prior distribution of parameters: (i) all prior distributions uniform (P-I) and (ii) distributions of effective population sizes and timing events log-uniform (P-II).

The presented results derive from pairwise ABC comparisons between 11 *Sinanodonta woodiana* populations with the best-supported evolutionary scenarios for each of 55 analyses.

The selected scenarios had the highest relative posterior probability with non-overlapping 95% Credible Intervals (CIs). In the case of more selected scenarios (with overlapping CIs), they are presented in order of decreasing posterior probability. Comparisons with three or more similarly supported scenarios (five or more in case of analyses with P-II priors) are referred to as "many". Perfect concordance in scenario choice between analyses with different priors in indicated in green, partial concordance in light green and complete disagreement in red. For population sample codes see Table 1.

|  | **BGIS** | | **ROSV** | | **ROMU** | | **CZKY** | | **CZTR** | | **PLSZ** | | **PLKO** | | **PLLI** | | **PLOP** | | **PLSP** | |
| --- | --- | --- | --- | --- | --- | --- | --- | --- | --- | --- | --- | --- | --- | --- | --- | --- | --- | --- | --- | --- |
|  | P-I | P-II | P-I | P-II | P-I | P-II | P-I | P-II | P-I | P-II | P-I | P-II | P-I | P-II | P-I | P-II | P-I | P-II | P-I | P-II |
| **ROSV** | 12, 11 | 11, 12 |  |  |  |  |  |  |  |  |  |  |  |  |  |  |  |  |  |  |
| **ROMU** | 7 | 7 | many | many |  |  |  |  |  |  |  |  |  |  |  |  |  |  |  |  |
| **CZKY** | 6 | 5, 6, 9, 1 | 8 | 8 | 8 | 8, 9, 6, 5 |  |  |  |  |  |  |  |  |  |  |  |  |  |  |
| **CZTR** | 6 | 6 | 5, 6 | 10, 5, 1, 6 | 8 | 8, 12, 3, 11 | 11, 6 | 6 |  |  |  |  |  |  |  |  |  |  |  |  |
| **PLSZ** | 6, 12 | many | 6 | 8 | many | many | 6, 4 | 6, 4, 10 | 6, 4 | 9, 6, 4, 1 |  |  |  |  |  |  |  |  |  |  |
| **PLKO** | 12 | 12 | 12 | 10, 12 | 8 | 8 | 4 | 4, 10 | 6 | 6 | many | many |  |  |  |  |  |  |  |  |
| **PLLI** | many | 11, 12 | 11 | 8 | 11, 8 | 8, 11, 12 | 11, 6 | 6, 11, 7 | 6 | 6 | 6 | 7, 12 | 11 | 11 |  |  |  |  |  |  |
| **PLOP** | 6 | 6, 11 | 8 | 8 | 8 | 8, 12 | 6 | many | 6 | 6, 12, 11, 8 | 5, 6 | 3, 8, 6 | 6 | 6 | 12 | 12 |  |  |  |  |
| **PLSP** | 6 | 8, 6 | 12 | 12, 8, 6, 10 | many | 8 | 11, 6 | 6 | many | 8, 6, 12 | many | 9, 6, 5 | 6 | 6 | 6 | 6 | 6, 11 | 8, 11, 6, 9 |  |  |
| **FR** | many | 2 | 11, 12 | 11 | 11, 12 | 2, 7, 11, 12 | 7 | 7 | many | 11 | 7, 2 | 2, 7 | 7 | 7, 11 | many | 2 | 7 | 7, 11, 2 | 7 | 7 |

**Table S6** Posterior error rates for each of 55 pairwise ABC comparisons (using a uniform prior distribution of parameters) between 11 European *Sinanodonta woodiana* populations estimated with DIYABC v 2.1.0.

|  |  |  |  |  |  |  |  |  |  |  |
| --- | --- | --- | --- | --- | --- | --- | --- | --- | --- | --- |
|  | BGIS | ROSV | ROMU | CZKY | CZTR | PLSZ | PLKO | PLLI | PLOP | PLSP |
| ROSV | 0.83 |  |  |  |  |  |  |  |  |  |
| ROMU | 0.91 | 0.88 |  |  |  |  |  |  |  |  |
| CZKY | 0.78 | 0.86 | 0.76 |  |  |  |  |  |  |  |
| CZTR | 0.82 | 0.87 | 0.78 | 0.79 |  |  |  |  |  |  |
| PLSZ | 0.86 | 0.86 | 0.85 | 0.81 | 0.86 |  |  |  |  |  |
| PLKO | 0.81 | 0.81 | 0.80 | 0.77 | 0.76 | 0.77 |  |  |  |  |
| PLLI | 0.82 | 0.73 | 0.85 | 0.77 | 0.87 | 0.81 | 0.75 |  |  |  |
| PLOP | 0.79 | 0.77 | 0.79 | 0.78 | 0.87 | 0.79 | 0.82 | 0.79 |  |  |
| PLSP | 0.79 | 0.83 | 0.77 | 0.78 | 0.79 | 0.78 | 0.75 | 0.85 | 0.81 |  |
| FR | 0.84 | 0.81 | 0.90 | 0.73 | 0.86 | 0.86 | 0.90 | 0.81 | 0.78 | 0.83 |
|  |  |  |  |  |  |  |  |  |  |  |

**Fig. S1** STRUCTURE output for K of 2-4 for 10 replicate runs based on 10 microsatellite markers and 369 individuals from 16 European and 137 individuals from 6 Chinese populations of *S. woodiana* summarized with CLUMPAK. Proportion of independent STRUCTURE runs generating the results presented are provided on the right. For sample codes see Table 1.

The STRUCTURE analysis was performed with admixture model, alleles correlated model and with no *a priori* information about location of samples. Ten independent runs per K included 300,000 burn-in iterations followed by 600,000 iterations.

**
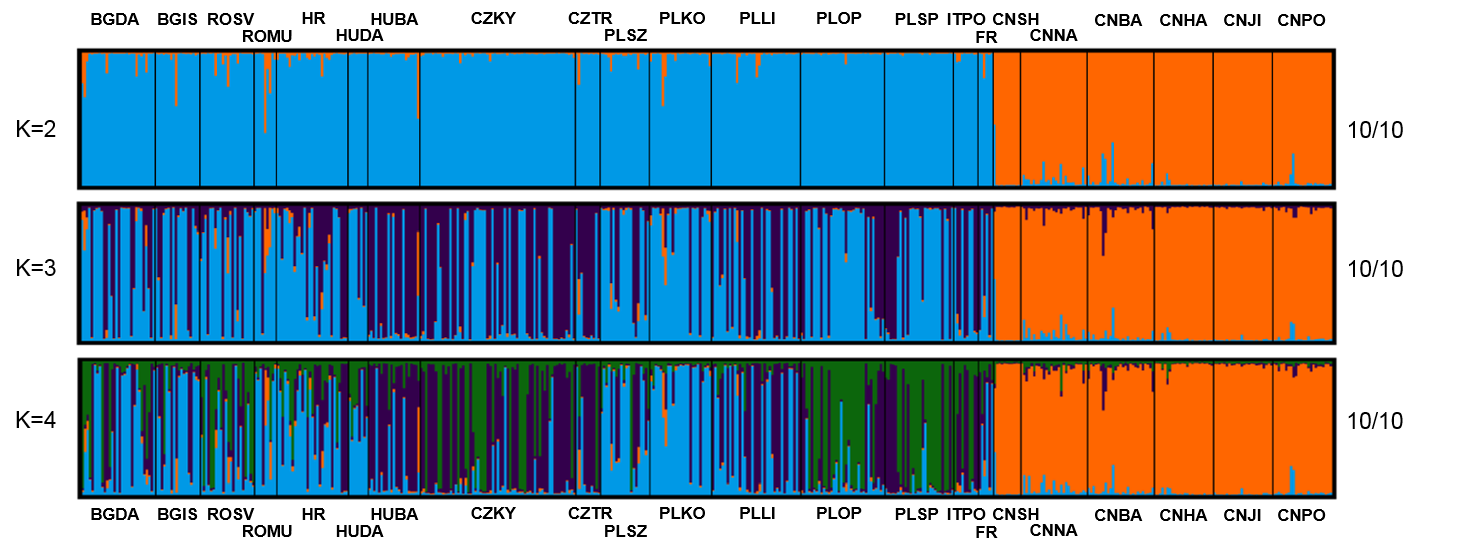
**

**Fig. S2** Description and graphical presentation of each of 12 evolutionary scenarios used in ABC pairwise comparisons in DIY ABC 2.0.4 (Cornuet et al., 2014).

The script that was used to design a simulation of each scenario is provided below each graph. The first line represents the simulated population samples: *F* is the parameter for the effective population size of the first member of the two contrasted population samples; *S* defines the same for the second member of the pair; *A* is the putative unsampled ancestral population; and *U* the putative unsampled population representing any other potential population serving in the foundation of any of the two sampled populations. The other parameters are as follows: *bF*, *bS* and *bU* are duration of bottlenecks, in number of generations, when the first, second and unsampled populations have the effective population size of *fF*, *fS* and *fU*, respectively. The parameter *t* stands for time of any demographical event (*varNe* is change of effective population size, *split* is admixture of two previously isolated populations creating a new descendant population, *merge* is divergence of one ancestral population to two descendant ones) in number of generations to the past. The parameter *r* is the admixture rate (proportion of genotypes originating from the first of the two ancestral populations during the merging event of two ancestral to a single descendant population (coded as *split*).

Between the temporal and effective population size parameters the following conditions were set:

tSzF<tFzA, tFzS<tSzA, tSzU<tUzA, tFzU<tUzA, tSx<tUzF, tSx<tFzA, tSx<tUzA, tSx<tFzU, tUzS<tSzA, tUzF<tFzA, tFx<tUzS, tFx<tSzA, tFx<tUzA, tFx<tSzU, bF<=tFzA, bF<=tFx, bF<=tFzS, bF<=tFzU, bS<=tSx, bS<=tSzU, bS<=tSzA, bS<=tSzF, bU<=tUzS, bU<=tUzA, bU<=tUzF, fF<=F, fS<=S, fU<=U.


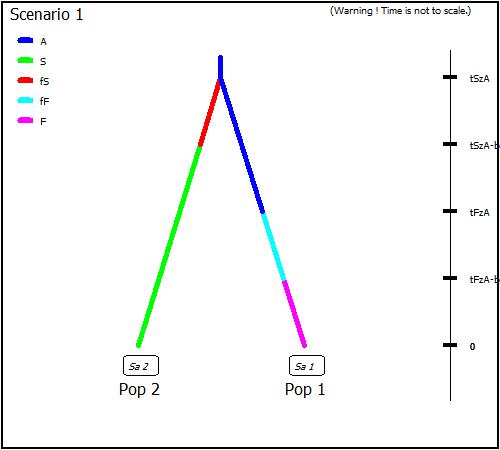

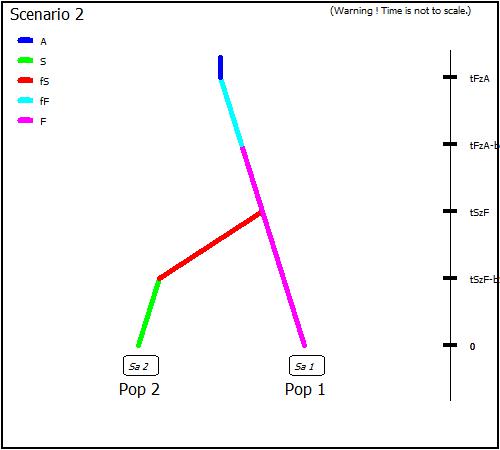


**Scenario 1**

F S A

0 sample 1

0 sample 2

tFzA-bF varNe 1 fF

tFzA merge 3 1

tSzA-bS varNe 2 fS

tSzA merge 3 2

**Scenario 2**

F S A

0 sample 1

0 sample 2

tSzF-bS varNe 2 fS

tSzF merge 1 2

tFzA-bF varNe 1 fF

tFzA merge 3 1

**Scenario 3**

F S A

0 sample 1

0 sample 2

tFzS-bF varNe 1 fF

tFzS merge 2 1

tSzA-bS varNe 2 fS

tSzA merge 3 2

**Scenario 4**

F S A U

0 sample 1

0 sample 2

tSzU-bS varNe 2 fS

tSzU merge 4 2

tUzA-bU varNe 4 fU

tUzA merge 3 4

tFzA-bF varNe 1 fF

tFzA merge 3 1


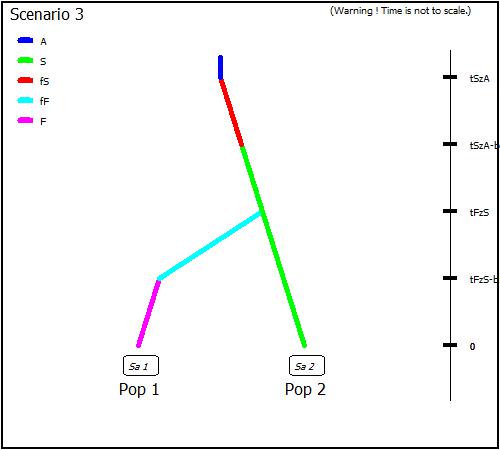

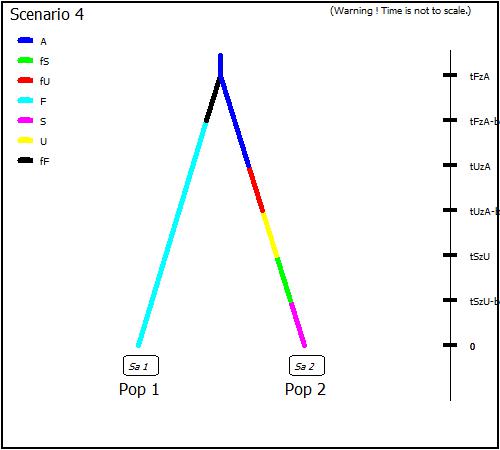


**Scenario 5**

F S A U

0 sample 1

0 sample 2

tFzU-bF varNe 1 fF

tFzU merge 4 1

tUzA-bU varNe 4 fU

tUzA merge 3 4

tSzA-bS varNe 2 fS

tSzA merge 3 2

**Scenario 6**

F S A U

0 sample 1

0 sample 2

tFzU-bF varNe 1 fF

tFzU merge 4 1

tSzU-bS varNe 2 fS

tSzU merge 4 2

tUzA-bU varNe 4 fU

tUzA merge 3 4

**Scenario 7**

F S A U

0 sample 1

0 sample 2

tSx-bS varNe 2 fS

tSx split 2 1 4 r

tUzF-bU varNe 4 fU

tUzF merge 1 4

tFzA-bF varNe 1 fF

tFzA merge 3 1

**Scenario 8**

F S A U

0 sample 1

0 sample 2

tFx-bF varNe 1 fF

tFx split 1 2 4 r

tUzS-bU varNe 4 fU

tUzS merge 2 4

tSzA-bS varNe 2 fS

tSzA merge 3 2


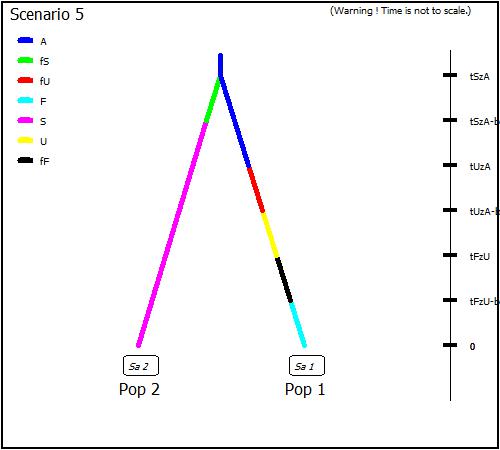

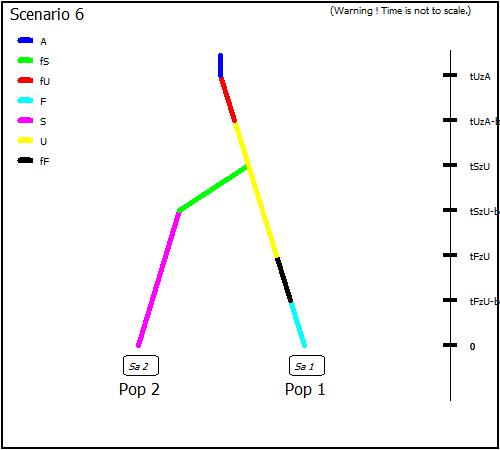

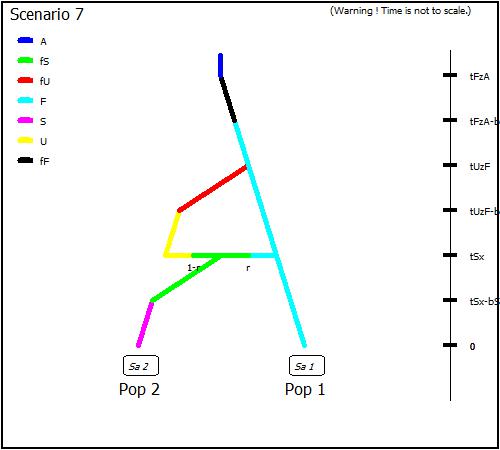

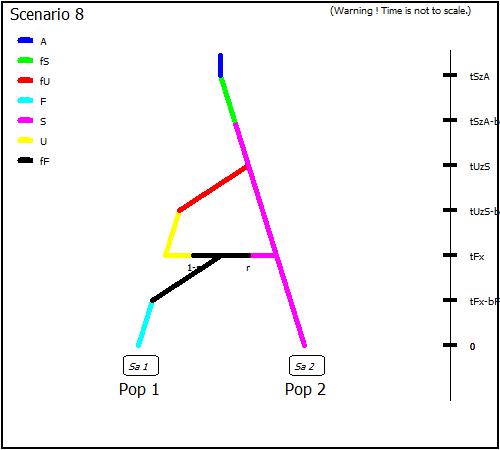


**Fig. S2** (continuation)

**Scenario 9**

F S A U

0 sample 1

0 sample 2

tFx-bF varNe 1 fF

tFx split 1 2 4 r

tUzA-bU varNe 4 fU

tUzA merge 3 4

tSzA-bS varNe 2 fS

tSzA merge 3 2

**Scenario 10**

F S A U

0 sample 1

0 sample 2

tSx-bS varNe 2 fS

tSx split 2 1 4 r

tUzA-bU varNe 4 fU

tUzA merge 3 4

tFzA-bF varNe 1 fF

tFzA merge 3 1

**Scenario 11**

F S A U

0 sample 1

0 sample 2

tFx-bF varNe 1 fF

tFx split 1 2 4 r

tSzU-bS varNe 2 fS

tSzU merge 4 2

tUzA-bU varNe 4 fU

tUzA merge 3 4

**Scenario 12**

F S A U

0 sample 1

0 sample 2

tSx-bS varNe 2 fS

tSx split 2 1 4 r

tFzU-bF varNe 1 fF

tFzU merge 4 1

tUzA-bU varNe 4 fU

tUzA merge 3 4


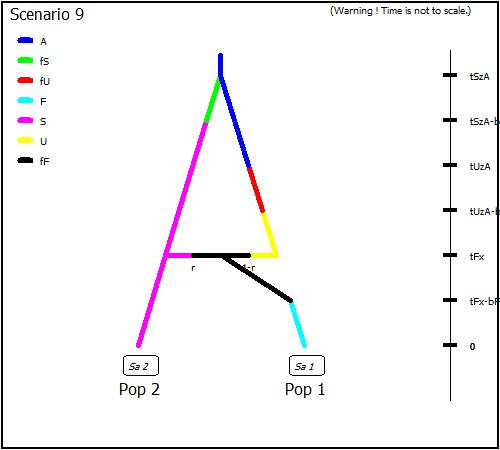

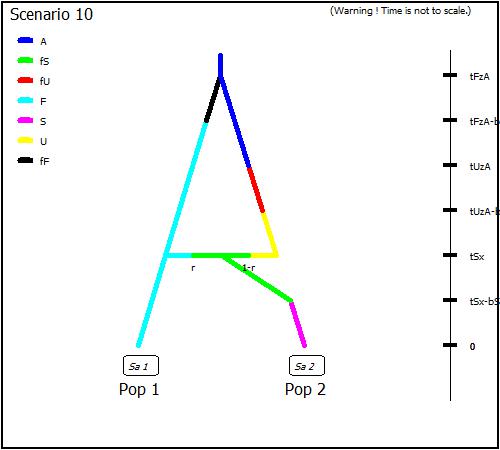

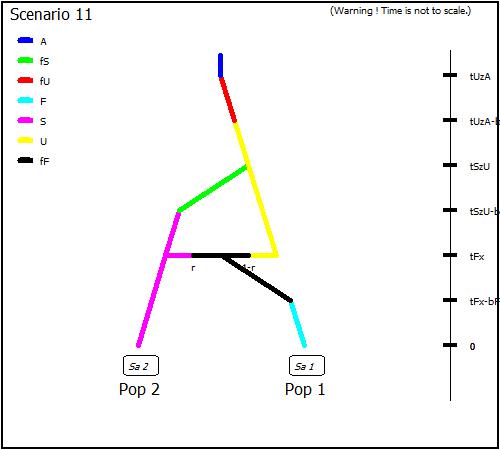

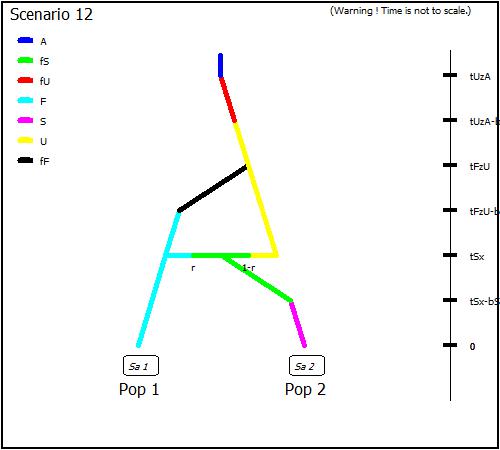


**Fig. S2** (continuation)

**Fig. S3** Graphic representation of logarithm of probability of data as a function of the number of assumed clusters (K).

Based on STRUCTURE version 2.3.4 (Pritchard et al., 2000) analysis of 369 individuals from 16 European populations. Twenty independent runs were performed for each value of K from 1 to 11.

**Fig. S4** Geographical distribution of microsatellite-marker genetic variation for all 16 European populations. Pie chart colours represent the proportional membership of individuals to microsatellite-based clusters for K=2, 5-8. Sample codes are summarized in Table 1.

**Fig. S4 (continuation)**


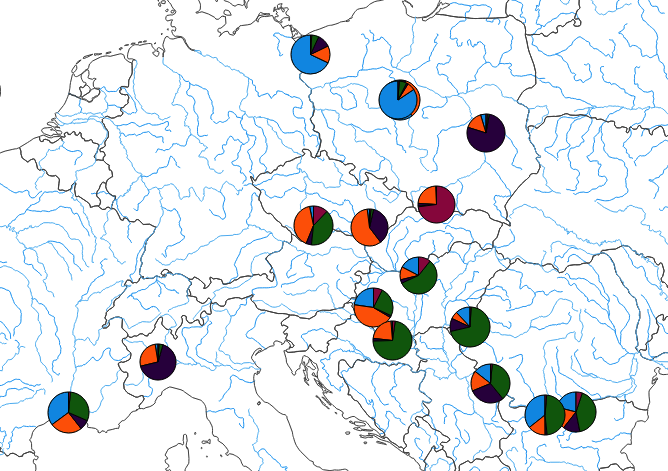


**FR**

**ITPO**

**HUBA**

**CZTR**

**CZKY**

**PLSZ**

**PLOP**

**PLSP**

**HUDA**

**HR**

**ROMU**

**ROSV**

**BGIS**


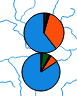


**BGDA**

**K = 5**

**PLLI**

**PLKO**


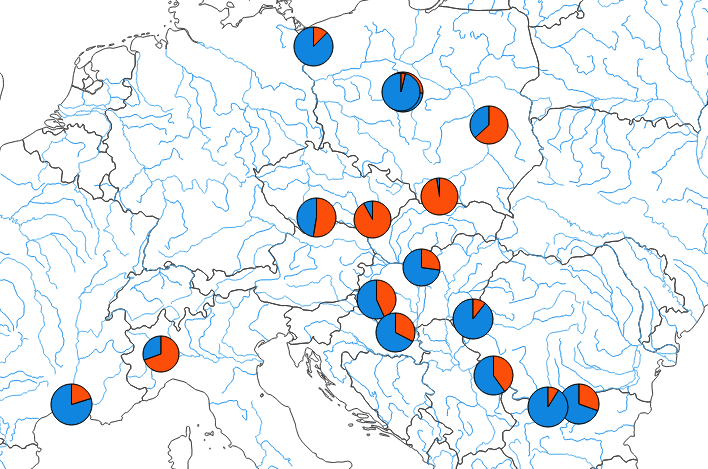

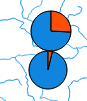


**PLLI**

**PLKO**

**FR**

**ITPO**

**HUBA**

**CZTR**

**CZKY**

**PLSZ**

**PLOP**

**PLSP**

**HUDA**

**HR**

**ROMU**

**ROSV**

**BGIS**

**BGDA**

**K = 2**


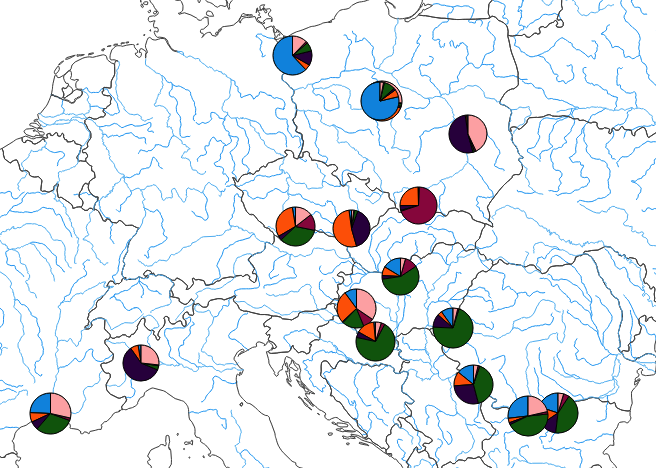


**FR**

**ITPO**

**HUBA**

**CZTR**

**CZKY**

**PLSZ**

**PLOP**

**PLSP**

**HUDA**

**HR**

**ROMU**

**ROSV**

**BGIS**

**BGDA**

**K = 6**


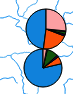


**PLLI**

**PLKO**


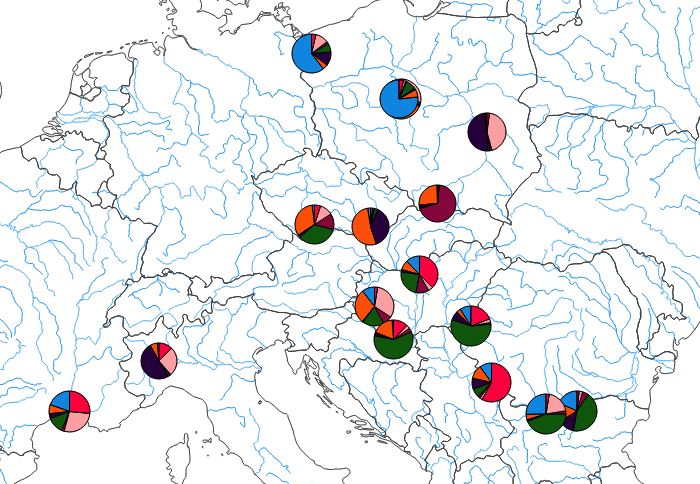

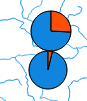


**FR**

**ITPO**

**HUBA**

**CZTR**

**CZKY**

**PLSZ**

**PLOP**

**PLSP**

**HUDA**

**HR**

**ROMU**

**ROSV**

**BGIS**

**BGDA**

**K = 7**


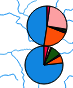


**PLLI**

**PLKO**

**Fig. S4 (continuation)**


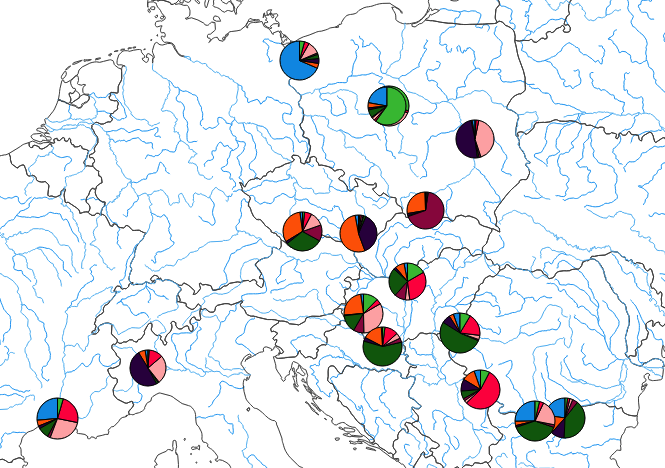


**FR**

**ITPO**

**HUBA**

**CZTR**

**CZKY**

**PLSZ**

**PLOP**

**PLSP**

**HUDA**

**HR**

**ROMU**

**ROSV**

**BGIS**

**BGDA**


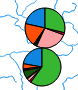


**K = 8**

**PLLI**

**PLKO**

**Fig. S5** Geographical representation of the most likely colonization pathways (indicated by arrows) of *S. woodiana* in Europe inferred by ABC pairwise comparisons between 11 populations (using a uniform prior distribution of parameters) and a putative ancestral source (A). The relationships are derived from the single winner scenarios and two cases of concordant equally supported scenarios. Red nodes refer to the source populations, blue nodes represent intermediate populations, and green nodes indicate derived populations.

Grey circles represent population samples that were not included in the ABC analyses. Yellow triangles indicate the geographic position of the oldest *S. woodiana* records in Europe. Red arrows indicate population samples that derived from a putative ancestral population by at least one pairwise comparison. The arrow size is proportional to strength of support. Dotted lines connect population samples whose pairwise ABC comparison resulted in most scenarios equally supported. Sample codes are given in Table 1.


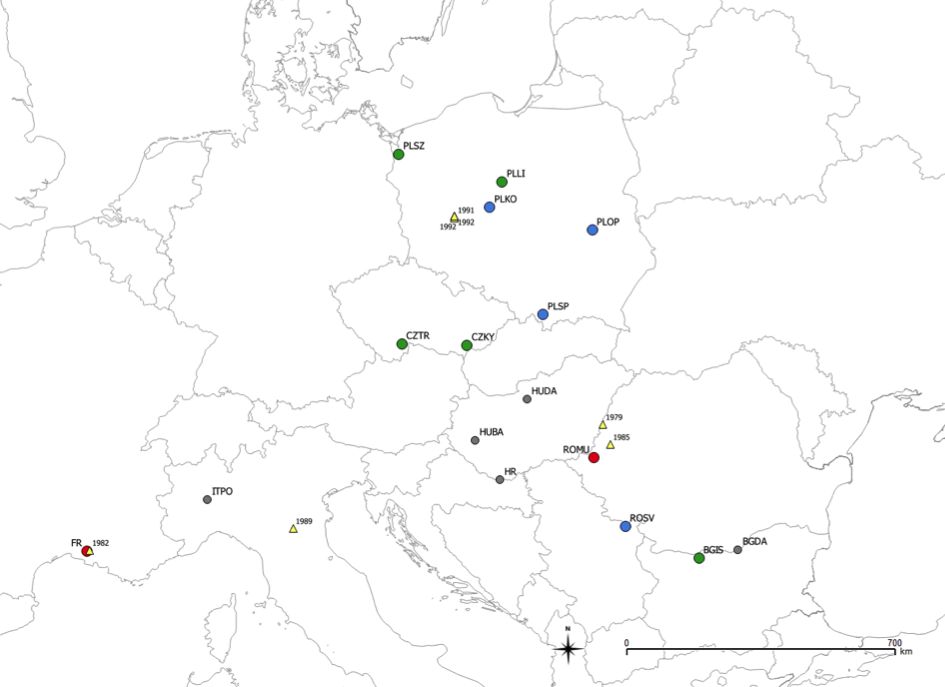


ANCESTRAL (A)

ANCESTRAL (A)

ANCESTRAL (A)

**Fig. S6** Model checking graphical representation for each of the 31 single-winner ABC pairwise comparisons (using a uniform prior distribution of parameters).

The graphs visualize the first four axes of PCA based on summary statistics and how well the observed dataset (yellow circle) fitted within the cloud of 1,000 datasets simulated under the most supported scenario (based on either posterior parameter distributions: larger circles, or prior distributions: smaller circles). For the list of the most supported scenarios for each comparison, see Tables S2 and S4. The model checking analyses were performed in DIYABC 2.0.4 (Cornuet et al., 2014). For population sample codes see Table 1.

The numerical representation of model checking analyses showed significant deviations of each observed summary statistic value from its distribution (on both sides out of 90 % of values) based on 1,000 simulated values drawn from posterior parameter distributions (according to the most supported scenario).

One of 32 observed summary statistic values fell significantly outside this range of simulated values (*P*<0.05 or *P*>0.95) in only six cases, two values in two cases, and four values in a single case:

comparison CZKY vs. BGIS - FST 1: p=0.975

comparison PLKO vs. ROSV - DM2 1: p=0.958

comparison PLLI vs. CZTR - MGW 1_1: p=0.0285

comparison PLLI vs. PLSZ - MGW 1_1: p=0.0240

comparison PLSP vs. PLKO - FST 2: p=0.9765

comparison CZKY vs. ROMU - FST 1: p=0.9555

comparison CZKY vs. ROSV - FST 1: p=0.9685 and DM2 1: p=0.978

comparison PLSZ vs. ROSV - DM2 1: p=0.956 and FST 2: 0.959

comparison PLKO vs. CZKY - MGW 1_1: p=0.0265, MGW 1_2: p=0.028, FST 1: p=0.977 and MGW 2_1: p=0.0235

**Fig. S6**

ROMU vs. BGIS


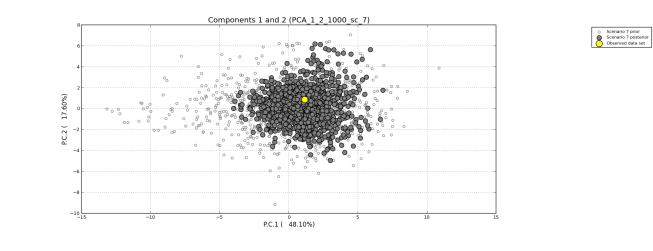

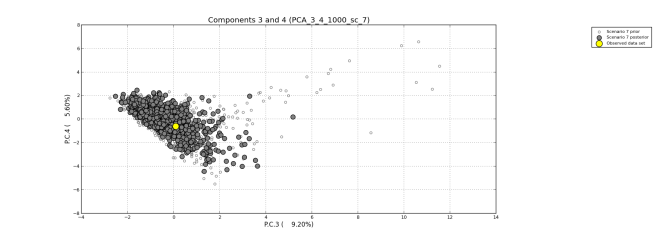


CZKY vs. BGIS


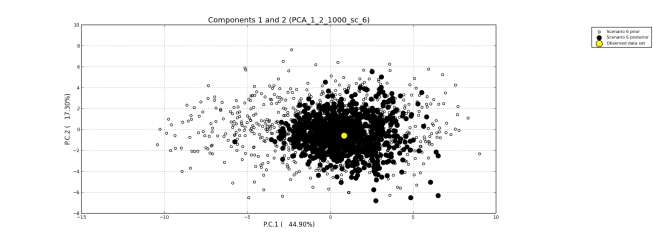

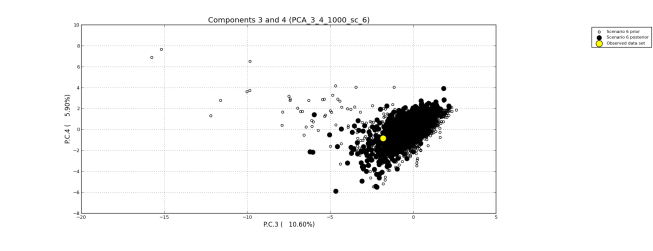


CZTR vs. BGIS


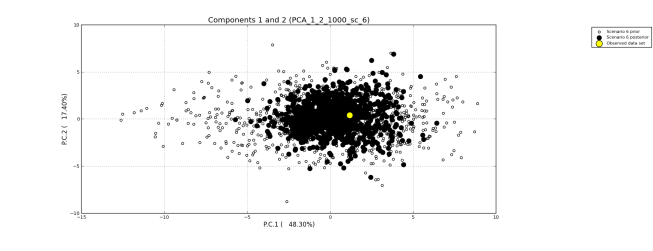

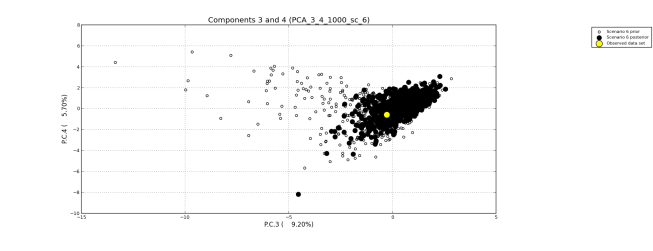


**Fig. S6** (continuation)

PLKO vs. BGIS


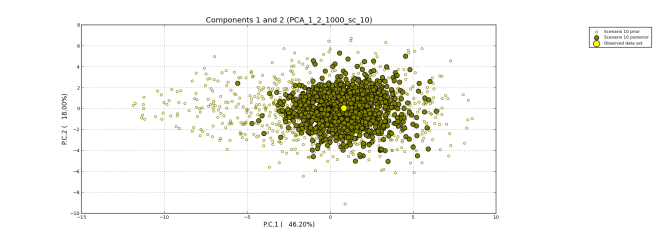

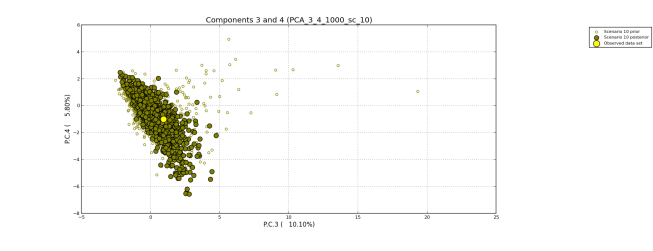


PLOP vs. BGIS


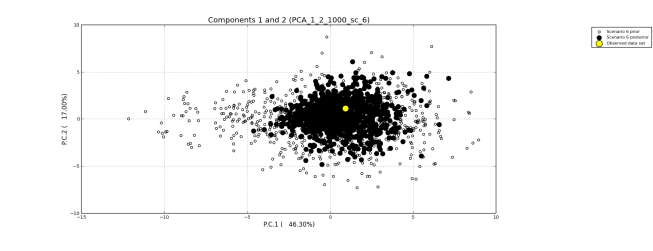

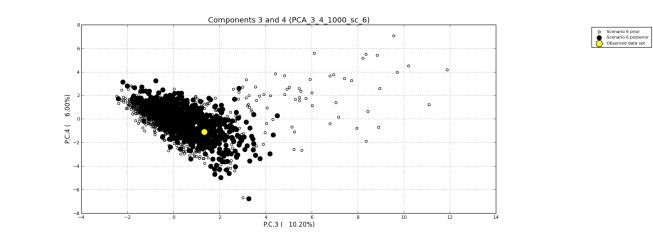


PLSP vs. BGIS


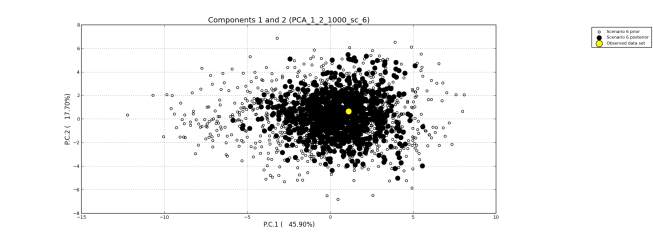

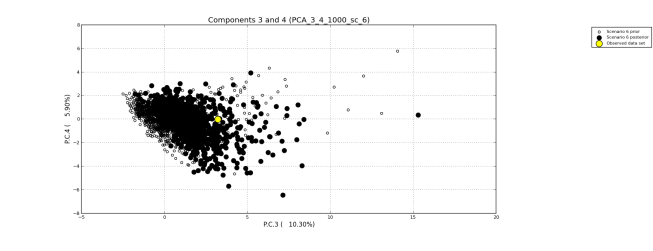


**Fig. S6** (continuation)

CZKY vs. ROSV


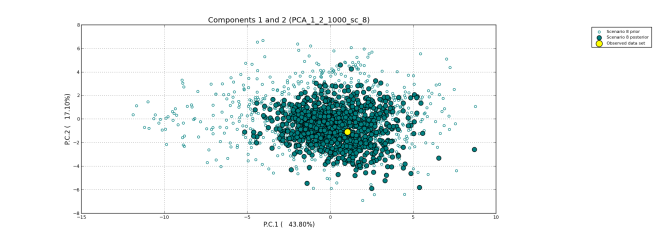

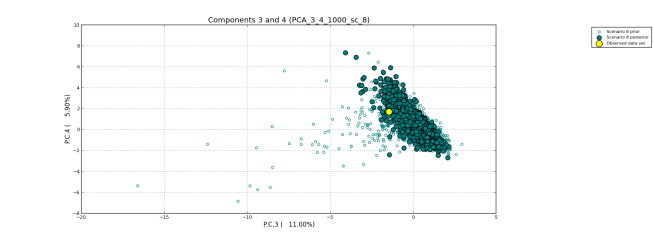


PLSZ vs. ROSV


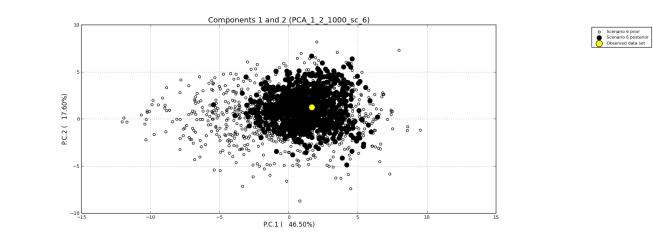

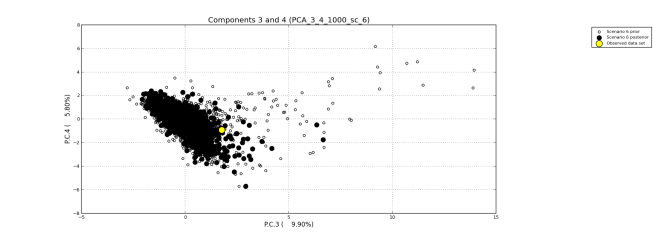


PLKO vs. ROSV


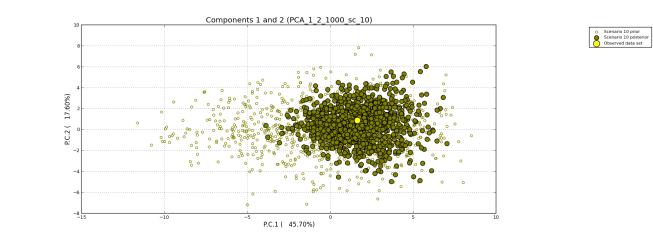

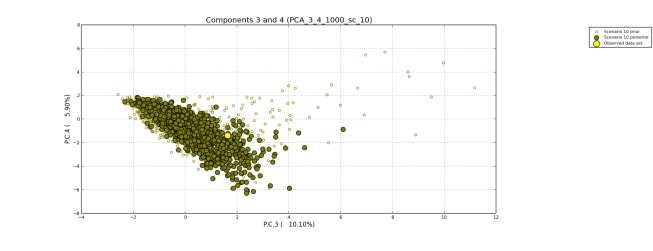


**Fig. S6** (continuation)

PLLI vs. ROSV


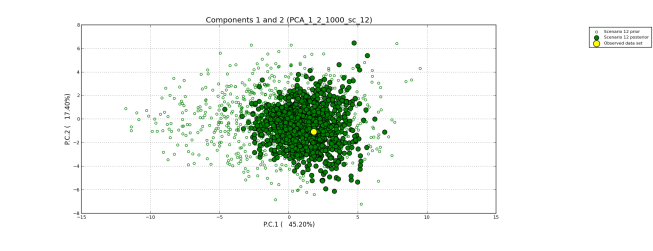

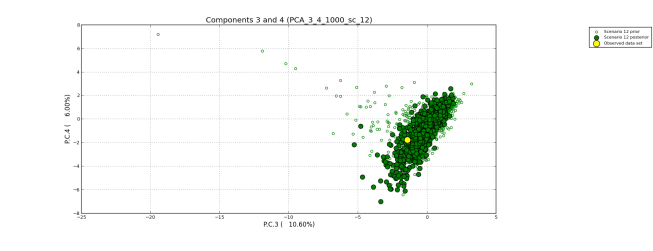


PLOP vs. ROSV


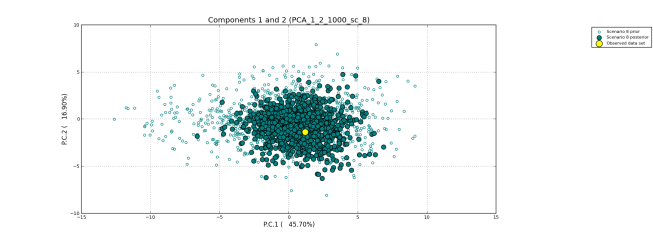

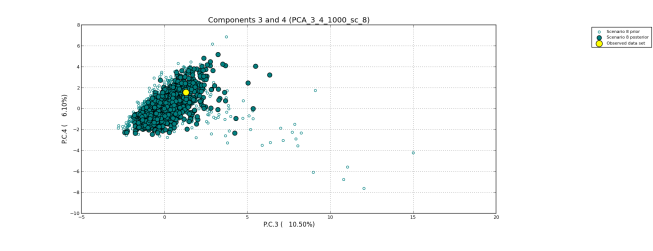


PLSP vs. ROSV


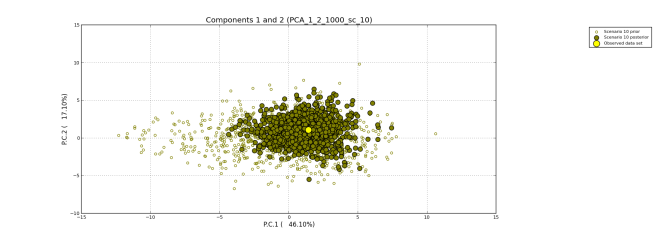

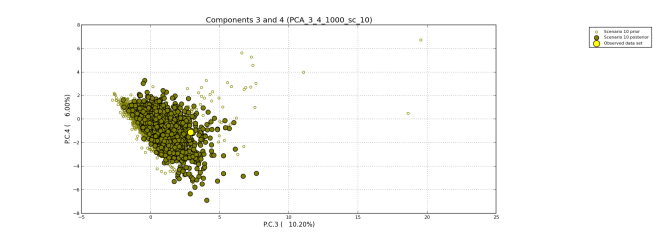


**Fig. S6** (continuation)

CZKY vs. ROMU


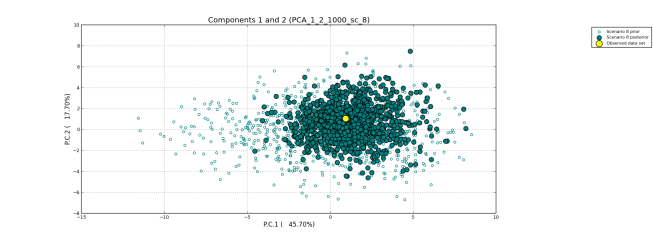

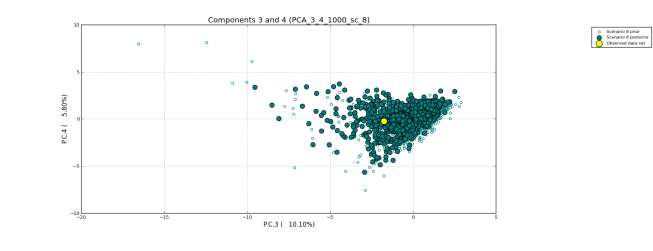


CZTR vs. ROMU


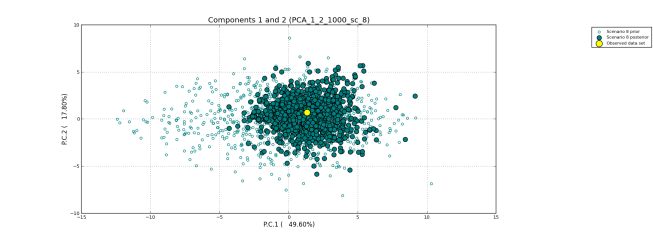

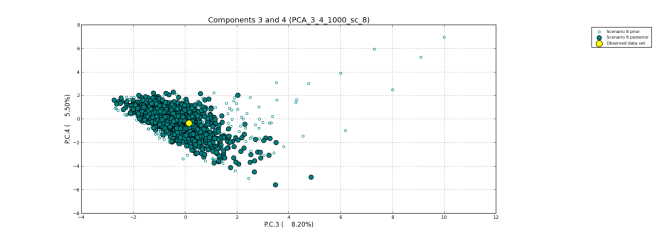


PLKO vs. ROMU


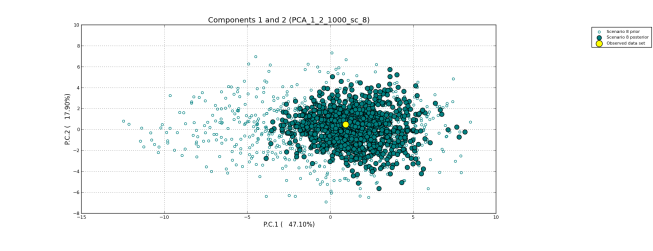

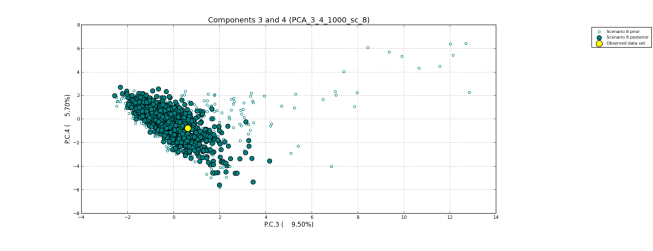


**Fig. S6** (continuation)

PLOP vs. ROMU


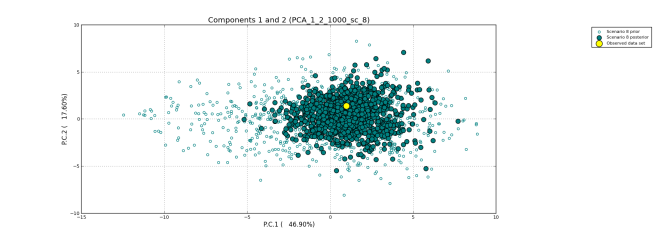

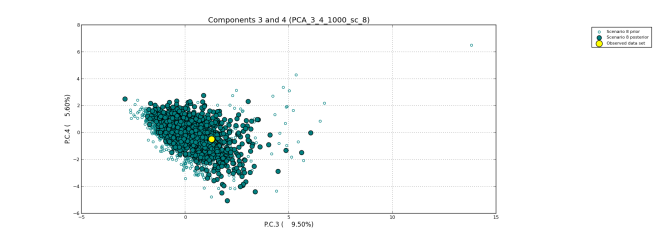


PLKO vs. CZKY


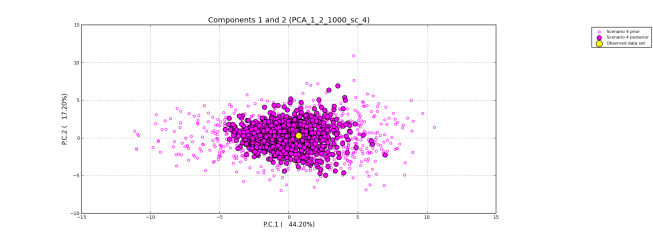

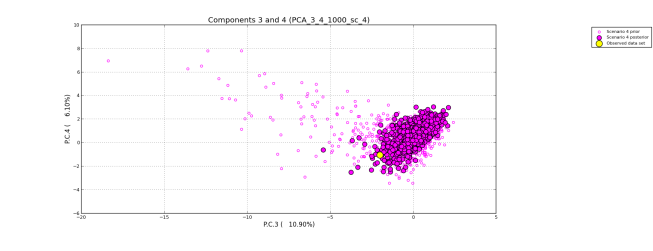


PLOP vs. CZKY


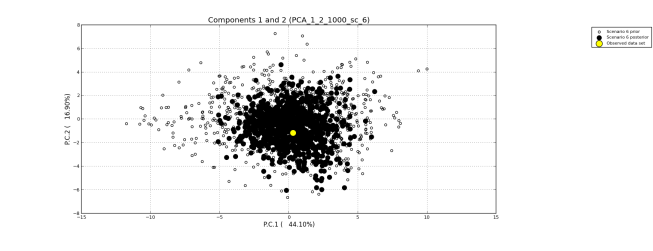

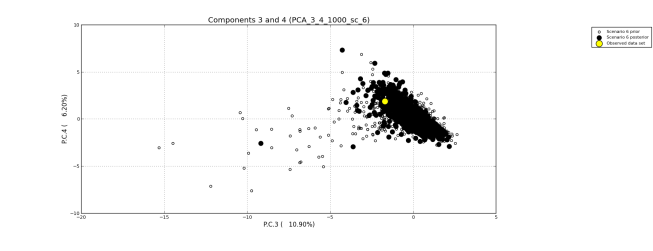


**Fig. S6** (continuation)

FR vs. CZKY


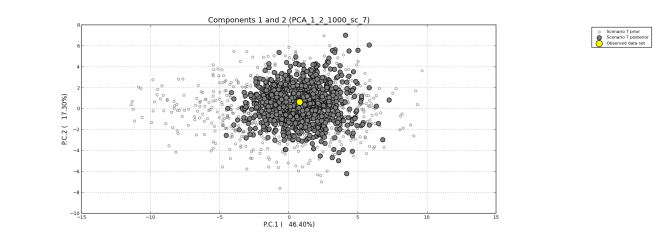

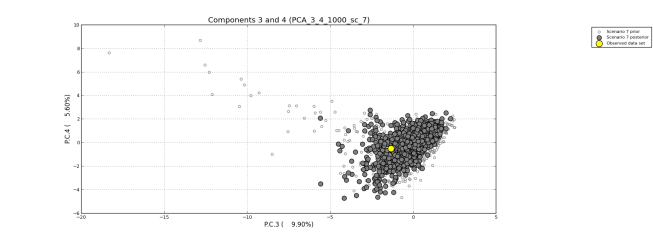


PLKO vs. CZTR


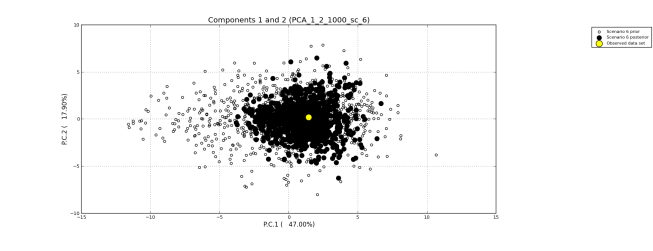

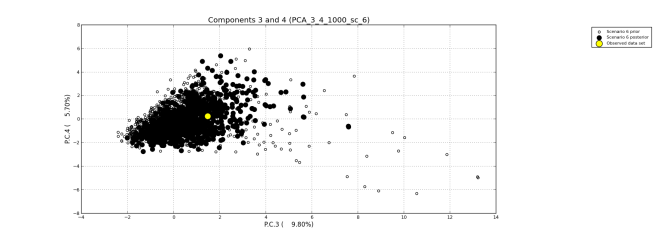


PLLI vs. CZTR


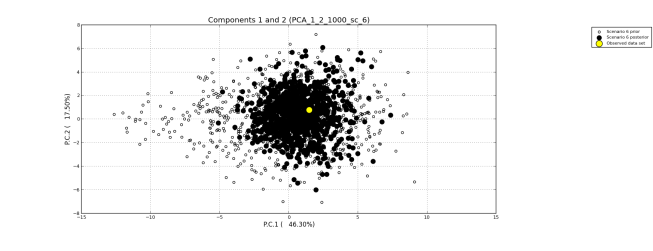

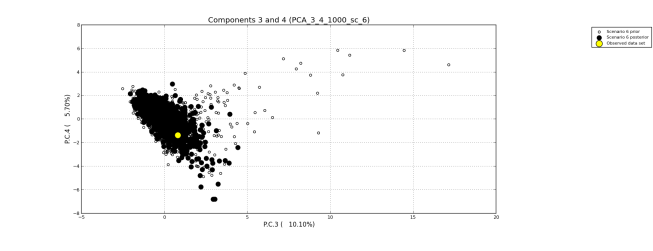


**Fig. S6** (continuation)

PLOP vs. CZTR


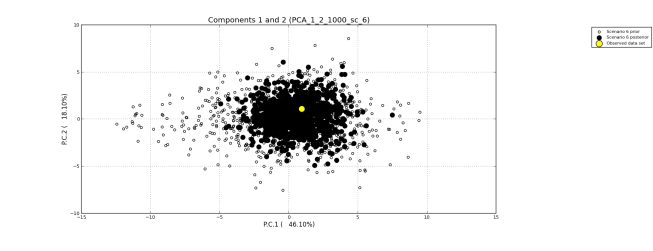

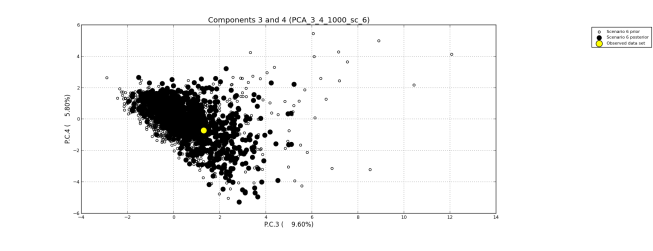


PLLI vs. PLSZ


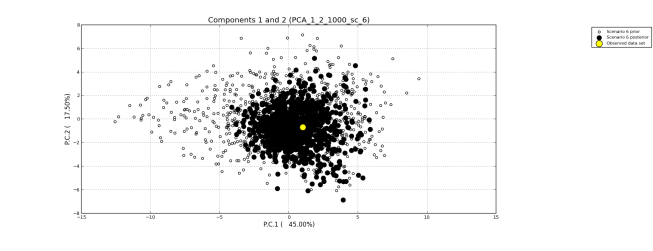

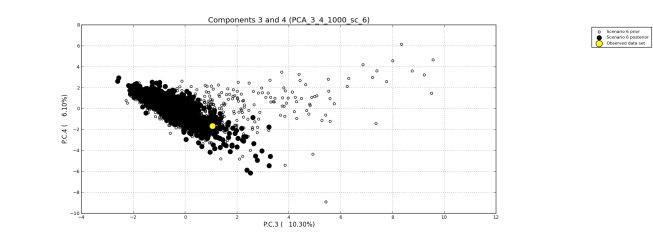


PLLI vs. PLKO


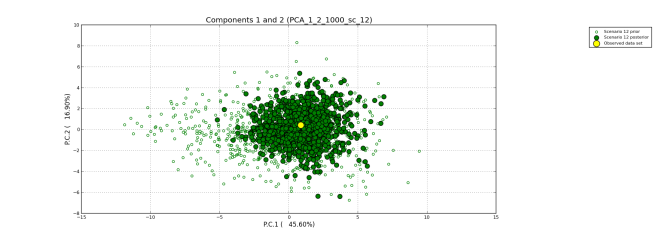

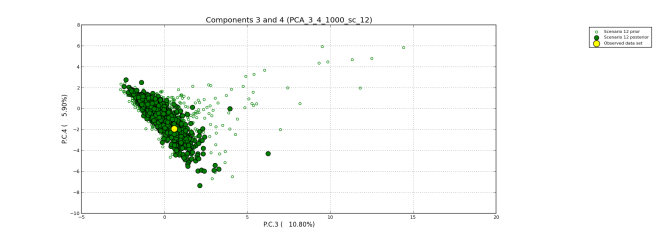


**Fig. S6** (continuation)

PLOP vs. PLKO


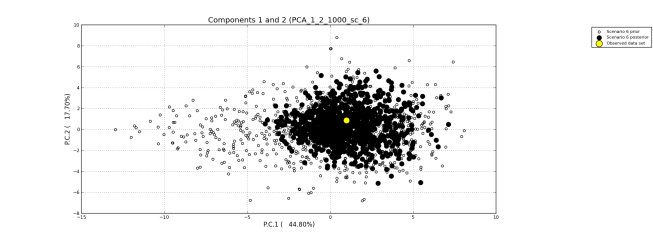

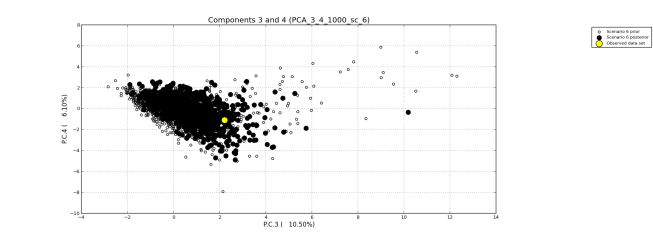


PLSP vs. PLKO


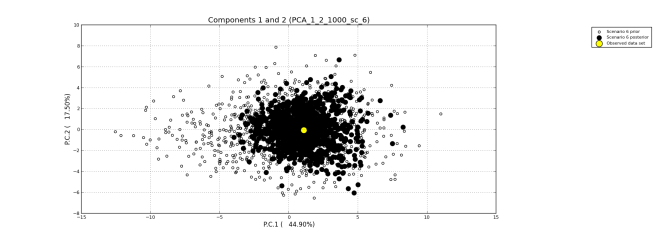

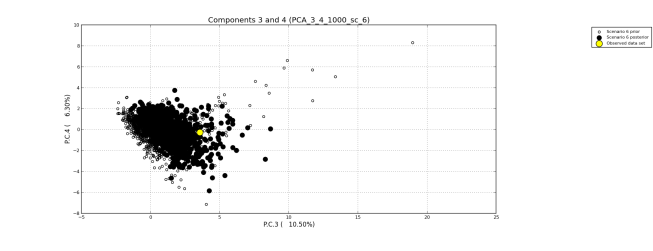


FR vs. PLKO


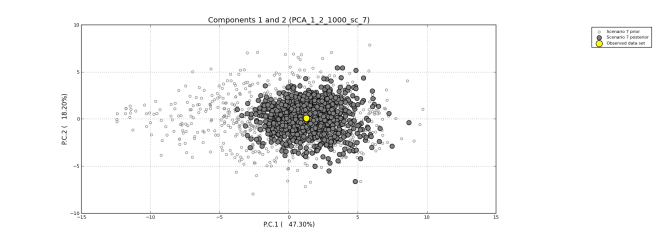

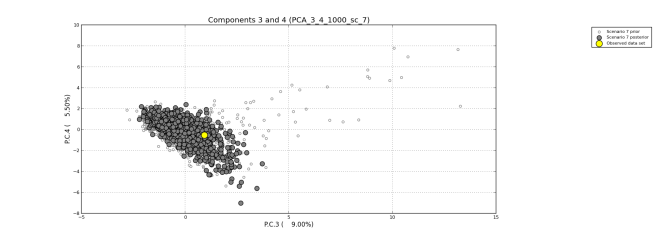


**Fig. S6** (continuation)

PLOP vs. PLLI


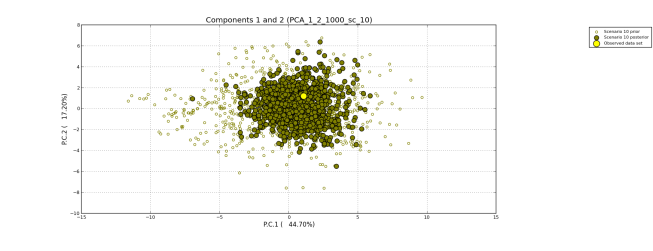

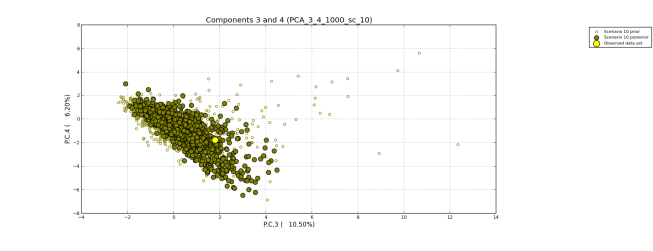


PLSP vs. PLLI


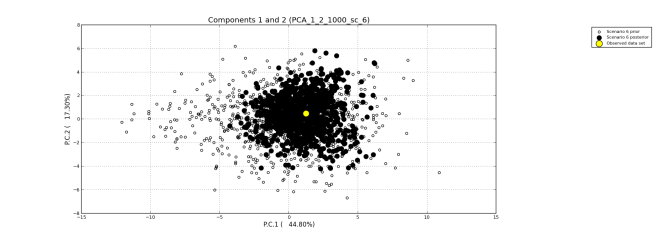

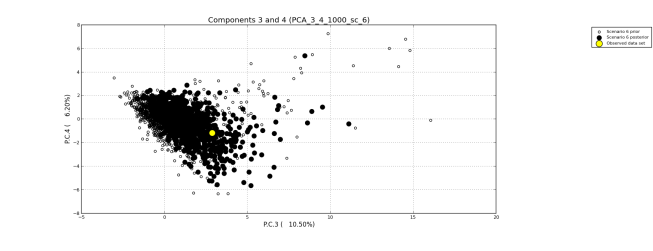


FR vs. PLOP


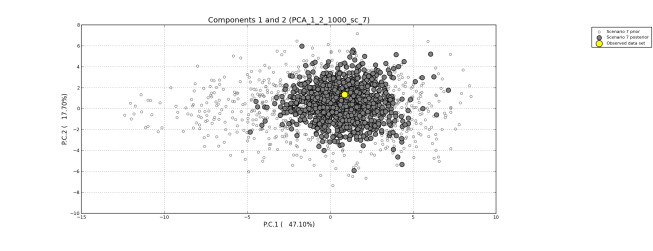

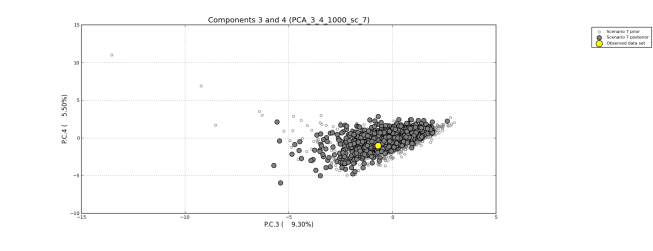


**Fig. S6** (continuation)

FR vs. PLSP


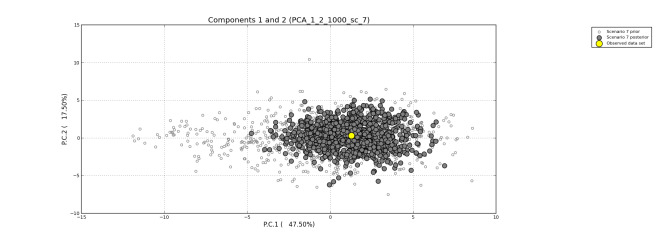

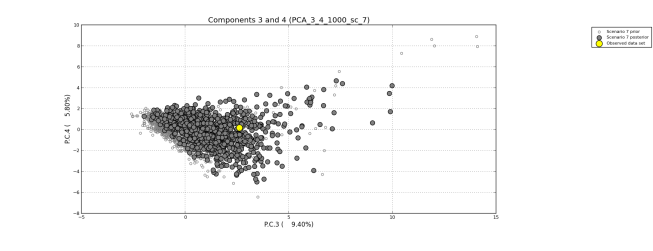

Supplement: Supplementary file 1 [file EVA-11-1975-s001.docx]
